# The association between the composition of the early-life intestinal microbiome and eczema in the first year of life

**DOI:** 10.3389/frmbi.2023.1147082

**Published:** 2023-03-16

**Authors:** Stefano Leo, Omer Faruk Cetiner, Laure F. Pittet, Nicole L. Messina, William Jakob, Laurent Falquet, Nigel Curtis, Petra Zimmermann

**Affiliations:** ^1^ Department for Community Health, Faculty of Science and Medicine, University of Fribourg, Fribourg, Switzerland; ^2^ Department of Paediatrics, Fribourg Hospital, Fribourg, Switzerland; ^3^ Istanbul Faculty of Medicine, Istanbul University, Istanbul, Türkiye; ^4^ Department of Paediatrics, The University of Melbourne, Parkville, VIC, Australia; ^5^ Infectious Diseases Research Group, Murdoch Children’s Research Institute, Parkville, VIC, Australia; ^6^ Pediatric Infectious Diseases Unit, Geneva University Hospitals and Faculty of Medicine, Geneva, Switzerland; ^7^ Microbiology Laboratory, Fribourg Hospital, Fribourg, Switzerland; ^8^ Department of Biology, University of Fribourg and Swiss Institute of Bioinformatics, Fribourg, Switzerland; ^9^ Infectious Diseases Unit, The Royal Children’s Hospital Melbourne, Parkville, VIC, Australia

**Keywords:** atopic dermatitis, allergies, microbiota, stool, intestine, shotgun metagenomic sequencing, infants, gut

## Abstract

**Introduction:**

The early-life intestinal microbiome plays a crucial role in the development and regulation of the immune system. Perturbations in its composition during this critical period have been linked to the development of allergic diseases.

**Objective:**

This study aimed to investigate the association between the composition of the early-life intestinal microbiome and the presence of eczema in the first year of life using shotgun metagenomic sequencing and functional analyses (metabolic pathways).

**Methods:**

Stool samples from 393 healthy term infants collected at 1 week of age were analyzed with shotgun metagenomic sequencing. Environmental and clinical data were prospectively collected using 3-monthly validated questionnaires. Participants were clinically assessed during study visits at 12 months of age. Eczema was diagnosed by the UK diagnostic tool and by a research nurse. Data analysis was stratified by delivery mode.

**Results:**

Eczema was diagnosed in 16.4% (60/366) of participants by nurse diagnosis. Infants born by cesarean section (CS) with nurse-diagnosed eczema had a higher relative abundance of *Escherichia, Shigella, Enterobacter*, and *Citrobacter* and a lower relative abundance of *Veillonella* than CS-born infants without eczema. In addition, CS-born infants without eczema had a higher abundance of genes involved in lactic fermentation. Vaginally born infants with eczema had a higher relative abundance of *Bacteroides* and a lower abundance of *Streptococcus.*

**Conclusion:**

There is an association between the bacterial composition of the intestinal microbiome at 1 week of age and the presence of eczema in the first 12 months of life.

## Graphical Abstract

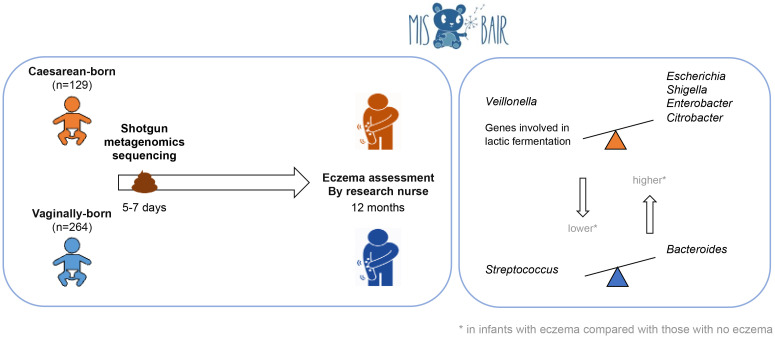

## Introduction

Atopic eczema, also called atopic dermatitis, is an inflammatory disease of the skin characterized by a variety of symptoms, including skin dryness, itchiness, reddish patches, and xerosis. Eczema is considered the first manifestation of the atopic march, and infants affected by eczema are more likely to suffer from other allergic diseases later in life (van der Hulst et al., 2007; Hill and Spergel, 2018; Sroka-Tomaszewska and Trzeciak, 2021). Furthermore, severe eczema is associated with the presence of other diseases, such as diabetes, cardiovascular and autoimmune diseases, and mental health disorders (Sroka-Tomaszewska and Trzeciak, 2021).

Infants are the age group most frequently affected by eczema (de Lusignan et al., 2021). Risk factors for eczema include being first born, being of Asian or black ethnicity, having a higher socioeconomic status, and living in an urban area (Penders et al., 2013; de Lusignan et al., 2021). The intestinal microbiome also plays a crucial role in the development and regulation of immune responses. Consistent with this, there is evidence that allergic diseases, such as eczema, are influenced by the composition of the intestinal microbiome (Zimmermann et al., 2019). As a critical step in developing immunotolerance, the immune system learns to recognize commensal microbiota. It has been hypothesized that reduced exposure to microbial immune stimulation, particularly *via* reduced microbiome diversity, impairs the healthy development of the immune system and increases the risk of allergic disease (Rook and Stanford, 1998). However, a recent systematic review suggests that particular microbes might be more important than bacterial diversity (Zimmermann et al., 2019). Children who develop eczema, allergic sensitization, or asthma have been reported to have a greater abundance of *Clostridiaceae* and *Enterobacteriaceae* and a lower abundance of *Lactobacillaceae* (Zimmermann et al., 2019). The findings reported from studies in neonates are more consistent than those reported from studies in older children and suggest that the early-life microbiome is crucial.

This is the first study to use shotgun metagenomic sequencing and functional analyses to investigate whether the composition of the early-life intestinal microbiome is associated with the prevalence and severity of eczema in the first year of life in a large cohort of infants. Understanding this relationship might help with the early detection of infants at risk of developing eczema and provide a basis for evidence-based interventions to prevent or correct dysbiosis of the microbiome.

## Methods

### Study design and participants

The participants were a subset of infants from the Melbourne Infant Study: BCG for Allergy and Infection Reduction (MIS BAIR) (Messina et al., 2019). In this randomized trial, 1,272 healthy infants were recruited antenatally to investigate whether Bacille Calmette–Guérin (BCG) immunization given in the first 10 days of life protects against childhood infection, allergy, and asthma. Inclusion criteria for infants were as follows: born after at least 32 weeks of gestation; birth weight greater than 1,500 g; absence of symptoms or signs of illness, including skin diseases; and no contraindication to BCG vaccination. Detailed data on demographics, pregnancy, and the perinatal period were collected at baseline. Three-monthly validated parent questionnaires were used to prospectively collect data on eczema-related symptoms, severity, and management. A visit by a study nurse took place when children were 12 months of age to clinically assess the incidence and severity of eczema.

### Eczema diagnostic tools

The presence of eczema in the first 12 months of life was evaluated with two different complementary methods: (1) the cumulative incidence of any eczema in the first 12 months of life assessed by the UK diagnostic tool using data from the 3-monthly questionnaires (UK diagnostic tool) (Williams et al., 1994a; Williams et al., 1994b); and (2) the point prevalence of research nurse-diagnosed eczema at 12 months of age assessed by clinical assessment using the SCORing Atopic Dermatitis (SCORAD) system (nurse diagnosis).

The UK diagnostic tool was adapted in accordance with the participants’ ages, as well as for online data collection (Pittet et al., 2022a). Eczema was defined as an itchy skin condition (major criterion) accompanied by at least three of the following minor criteria: (1) generally dry skin; (2) lesions in skin creases or cheeks; (3) atopic disease in a first-degree relative; and (4) visible eczema involving the flexures, head, or limbs. Previous data show that parents are able to accurately report eczema in their infants (Fleming et al., 2001).

In the absence of an agreed tool to reliably diagnose eczema in infancy (Pittet et al., 2022b), the use of two complementary tools improves the reliability of the results. Although the point prevalence estimate by a trained professional (nurse diagnosis outcome) is the most robust assessment, it captures lesions present on the day of visit only, depends on the management of eczema (i.e., effective treatment will mean lesions are absent), may miss seasonal fluctuation of eczema, and could capture lesions that are not necessarily atopic dermatitis (Pittet et al., 2022b). Being widely used, the UK diagnostic tool enables comparison with other studies; however, it is less relevant for infant eczema, with one of the major limitations being that the score requires the subject to experience itching (which excludes infants who are not yet developmentally capable of scratching themselves). In addition, the minor criteria are primarily based on flexural lesions, and not on the typical distribution of eczema in infancy (i.e., trunk and face, with only the latter included in the modified version) (Pittet et al., 2022b).

Age at onset of eczema, use of topical steroids, and eczema severity were also evaluated. Eczema severity was assessed using the Patient-Oriented Eczema Measure (POEM) score (Charman et al., 2004) included in each 3-monthly questionnaire and the SCORAD score no author, (no author, 1993) assessed by the research nurse at the study visit at 12 months.

### Stool collection

Parents were asked to collect stool samples from their infants in sterile tubes on each of the first 10 days of life. To minimize variation, parents were asked to collect from the first bowel movement of the day (with date and time recorded) and immediately freeze the stool in their domestic freezers (–18°C or below). Samples were collected from parents' homes by a study team member and kept frozen during transportation to the laboratory, where they were aliquoted for storage at –80**°**C. One stool sample collected between days 5 and 7 of life was analyzed for each infant.

### DNA extraction and shotgun metagenomic sequencing

Samples were thawed at room temperature and 100 mg of stool was mixed with 1x phosphate-buffered saline (PBS). DNA was extracted using the FastDNA™ SPIN Kit for Soil (MP Biomedicals, Illkirch-Graffenstaden, France). Of this stool suspension, 300 µl was mixed with 978 µL of PBS (provided by the kit) and 122 µL of MT buffer and transferred into a lysing matrix E (LME) tube. Sample homogenization was performed with the FastPrep96 homogenizer using the program for human stool samples (settings: 1,600 rpm, 40 s). The LME tubes were centrifuged at 14,000 g for 10 minutes and the supernatant was mixed with 250 µL of protein precipitation solution in a 2-mL microcentrifuge tube. The tubes were inverted by hand 10 times and centrifuged at 14,000 g for a further 5 minutes. Subsequently, 1 mL of binding matrix solution was then added to the supernatant. The tubes were inverted for 2 minutes, then rested for 5 minutes, and 800 µL of the supernatant was removed. The remaining supernatant was resuspended in the binding matrix. At this point, 700 µL was transferred into a new spin filter tube and centrifuged for 1 minute at 14,000 g. This step was repeated twice. After this, 500 µL of ethanol-based washing solution (SEWS-M wash buffer) was mixed with the binding matrix. Samples were centrifuged three times for 1 minute at 14,000 g and allowed to dry at room temperature for 5 minutes. DNA was eluted in 50 µL of DNase- and pyrogen-free water.

The DNA concentrations of samples and a negative control (i.e., no sample DNA) were measured using Qubit™ dsDNA High Sensitivity Assay kits (Life Technologies, CA, USA). The mean DNA concentration in samples was 367 ng; the negative control was below the detection limit (i.e., < 0.01 ng/µL) and was therefore not sequenced. Sequencing libraries were prepared with the Nextera DNA Flex library preparation kits (Illumina, San Francisco, CA, USA) to obtain insert sizes of approximately 600 bp. Illumina PhiX DNA was added to the libraries, which were then sequenced (2x149) on a NextSeq 550 system (Illumina) with high-output flow cells. Bacterial and fungal evenly distributed mock communities (Gut Microbiome Whole Cell Mix MSA-2006™ and Mycobiome Whole Cell Mix^TM^, respectively; ATCC, Manassas, VA, USA) were sequenced as positive controls.

### Pre-processing, taxonomic assignment, and relative abundance computation

The overall quality of the sequencing run was inspected with the Illumina Sequencing Analysis Viewer v. 2.4.7. The quality of the raw reads was assessed by FastQC v. 0.11.7 (http://www.bioinformatics.babraham.ac.uk/projects/fastqc/) and scanned with Trimmomatic v. 0.39 by using a sliding window of 20 nt (Bolger et al., 2014). Reads with an average quality below a Phred score of 28 and with a length of less than 100 nt (before or after trimming) were filtered out. Quality-filtered reads were then analyzed with Kraken v. 2.0.9-beta (Wood et al., 2019) and sequences mapping to the human (vGRCh38.p11) genome with a confidence score of more than 0.1 were discarded. The remaining reads were assigned to bacterial, archaeal, viral, and fungal genomes with Kraken2 (with a confidence score of more than 0.1) and with MetaPhlAn v. 3.0.7-1 (Segata et al., 2012). Compared with Kraken2, MetaPhlAn 3 uses a smaller reference sequences database, made up of clade-specific gene markers. Data obtained by MetaPhlAn 3 were used to confirm the results from the Kraken2/Bracken pipeline.

Read counts obtained with Kraken2 were corrected with Bracken v. 2.6.2 (Wood et al., 2019; Lu and Salzberg, 2020) at phylum, family, genus, and species levels. All the reference genome sequences were collected and analyzed with kmer2read_distr [settings: k-mer length (-k) of 35 and read length (–l) of 100] to finally generate a k-mer distribution file with generate_kmer_distribution.py. Eventually, the read abundance of each phylum, family, genus, and species in each sample was estimated with est_abundance.py (settings: minimum number of reads necessary for taxonomic classification (–t) = 10).

For each taxonomic level, the relative abundance was computed as a percentage by dividing the number of Bracken-corrected read counts in a given taxon by the total number of Bracken-corrected reads assigned to that taxonomic level in any given sample. MetaPhlAn 3 provided taxa and relative abundance already expressed as percentages.

### Phages

The phage content was investigated by selecting reads assigned to viruses from the *Caudovirales* order. *Caudovirales* includes double-stranded DNA phages from the families *Siphoviridae*, *Myoviridae*, and *Podoviridae*, which are the most abundant bacteriophages present in the human intestine (Hoyles et al., 2014; Shkoporov et al., 2019). The relative abundance of phages was computed by dividing the number of reads mapped to phage genomes by the total number of quality-filtered no-human reads. The ratio was then multiplied by 1,000,000 and the relative abundance was expressed as counts per million (CPM).

### Metabolic pathway analyses

Microbial metabolic pathways were identified by analyzing quality-filtered non-human reads with the HUMAnN v. 3.5 tool with default parameters (Beghini et al., 2021). Forward and reverse read files from each sample were concatenated in a unique FASTQ file. UniRef90 was used as a database. Normalized gene copies per million were then obtained from *pathabundance* output tables with *human_renorm_table* HUMAnN 3 code. The interpretation of metabolic pathways and their putative presence in each species were investigated by querying the MetaCyc database (https://biocyc.org/) (Caspi et al., 2006).

### Reference databases

The human genome (v. GRCh38.p11) and 15,009 bacterial, 511 archaeal, 11,833 viral, and 420 fungal genomes were downloaded from NCBI RefSeq on 3 March 2022.

### Statistical analysis

All statistical analyses were performed with the R software v. 4.2.0. The codes used for statistical analyses are reported in **Supplementary File 1**.

To compare the bacterial compositions of the stool microbiomes at the species and genus level, principal coordinate analyses (PCoAs) were performed. For the PCoAs, Bray-Curtis dissimilarity distance was computed on the genera or species’ relative abundance after square-root transformation with the *vegdist* function of the vegan R package v. 2.6-2. The resulting data were analyzed with the *betadisper* function (vegan). Differences in bacterial composition according to a given variable were assessed at the species, genus, family, and phylum taxonomic levels by permutational multivariate analysis of variance (PERMANOVA) tests. PERMANOVA was performed with the *adonis2* function (vegan) using Bray–Curtis dissimilarity distance computed on square root-transformed data and using 9,999 permutations.

Ecological indices, richness, and Shannon diversity were computed after rarefying read counts analyzed with the Kraken2/Bracken pipeline to 35,000 with the *rrarefy* function (vegan). Shannon indexes were computed with the *diversity* function (vegan).

To identify differentially abundant species, DESeq2 (Love et al., 2014) v. 1.36.0 and linear discriminant analysis of effect size [LEfSe (Segata et al., 2011)] as implemented in the microbiomeMarker package v. 1.2.2 were performed with the following settings: wilcoxon_cutoff = 0.05, norm = “CPM”, kw_cutoff = 0.05, lda_cutoff = 2, and enrich_group = 0.

Prior to running differentially abundant analyses, we filtered out genera and species identified with the Kraken2/Bracken pipeline that had less than 10 reads in less than 100 (25.5%) of 393 samples. LEfSe was also used to identify differentially abundant pathways with the same settings as described above, except the lda_cutoff was set at 1.5. For all other statistical testing, the Wilcoxon rank-sum test was used. Results were considered significant when associated *p*-values (or false-discovery-rate-adjusted *p*-values for DESeq2 analyses) were less than 0.05. Plotting was performed with the ggplot2 package v. 3.3.6.

As delivery mode was expected to have a strong effect on the intestinal microbiome (Zimmermann and Curtis, 2018), data analysis was stratified according to delivery mode. Further factors investigated for their effect on the composition of the intestinal microbiome included infant (sex, ethnicity, gestational age, birth weight, feeding method, and antibiotic exposure), maternal (antibiotic use during the last trimester of pregnancy or during labor, and smoking), environmental (number of siblings, childcare attendance, number of smokers in the household, and animal exposure during pregnancy or at 12 months of age), and familial [family history of atopic disease (defined as eczema, hay fever, or asthma) or allergies (food and/or secondary allergies, eczema, hay fever, and/or asthma)] factors.

### Ethics

Informed consent was obtained from participants’ parents or guardians. The study was approved by the Royal Children’s Hospital Human Research Ethics Committee (HREC, authorization 53543). MIS BAIR is registered with the Australia and New Zealand Clinical Trials Registry (1051228) and the US National Institutes of Health (NCT01906853).

## Results

Of a total of 393 included term-born infants, 32.8% (129/393) were born by cesarean section (CS). Characteristics of the study cohort are summarized in **Table 1**. All four 3-monthly questionnaires were completed for 91.6% (360/393) of infants, and 93.1% (366/393) of infants attended the 12-month visit, with a median age of 13.3 months [interquartile range (IQR) 12.8–13.9 months]. Eczema was diagnosed in 30.6% (110/360) and 16.4% (60/366) of infants by the UK diagnostic tool and nurse diagnosis, respectively (**Table 2**).

**Table 1:** Participant characteristics and exposures during first year of life.

Factor	Total (*n* = 393), *n* (%) or median (IQR)	Cesarean section born (*n* = 129), *n* (%) or median (IQR)	Vaginally born (*n* = 264), *n* (%) or median (IQR)
Infant			
Sex, female	197 (50.1)	61 (47.3)	136 (51.5)
Ethnicity			
Caucasian (3 or 4 grandparents)	309 (78.6)	104 (80.6)	205 (77.7)
Asian (3 or 4 grandparents)	17 (4.3)	6 (4.7)	11 (4.2)
Mixed Caucasian and Asian	19 (4.8)	4 (3.1)	15 (5.7)
Other	48 (12.2)	15 (11.6)	33 (12.5)
Gestational age, weeks	39.3 (38.4–40.3)	39.0 (38.3–39.6)	39.6 (38.5–40.5)
Birth weight, g	3,440 (3,126–3,750)	3,440 (3,025–3,750)	3,450 (3,147.5–3760)
Formula milk before 7 days of life	129 (32.8)	61 (47.3)	68 (25.8)
Antibiotic exposure in first 12 months of life			
0–3 months	14 (3.6)	4 (3.1)	10 (3.8)
3–6 months	32 (8.1)	19 (14.7)	13 (4.9)
6–9 months	53 (13.5)	17 (13.2)	36 (13.6)
9–12 months	84 (21.4)	29 (22.5)	55 (20.8)
Maternal			
Smoking during pregnancy	10 (2.5)	6 (4.7)	4 (1.5)
Antibiotic use during last trimester of pregnancy	49 (12.5)	18 (14)	31 (11.7)
Antibiotic use during labor	90 (22.9)	21 (16.3)	69 (26.1)
Environmental			
Number of siblings			
0	196 (49.9)	53 (41.1)	143 (54.2)
1	135 (34.4)	52 (40.3)	83 (31.4)
2	48 (12.2)	19 (14.7)	29 (11.0)
3	8 (2.0)	4 (3.1)	4 (1.5)
>3	6 (1.5)	1 (0.8)	5 (1.9)
Childcare attendance in first 12 months of life			
0–3 months	9 (2.3)	5 (3.9)	4 (1.5)
3–6 months	26 (6.6)	8 (6.2)	18 (6.8)
6–9 months	88 (22.4)	33 (25.6)	55 (20.8)
9–12 months	168 (42.7)	55 (42.6)	113 (42.8)
Animal exposure during pregnancy			
Cats	104 (26.5)	38 (29.5)	66 (25)
Dogs	140 (35.6)	50 (38.8)	90 (34.1)
Other pets	55 (14)	23 (17.8)	32 (12.1)
Livestock	10 (2.5)	5 (3.9)	5 (1.9)
Animal exposure at 12 months of age			
Cats	98 (24.9)	37 (28.7)	61 (23.1)
Dogs	133 (33.8)	47 (36.4)	86 (32.6)
Other pets	52 (13.2)	21 (16.3)	31 (11.7)
Livestock	10 (2.5)	4 (3.1)	6 (2.3)
Familial			
Atopic disease,* any parent and/or sibling	340 (86.5)	112 (86.8)	228 (86.4)
Allergies,** any parent and/or sibling	335 (85.2)	110 (85.3)	225 (85.2)
Atopic disease,* both parents	116 (29.5)	36 (27.9)	80 (30.3)

IQR, interquartile range.

* eczema, hay fever, and/or asthma.

** food- and/or secondary allergies, eczema, hay fever, and/or asthma.

**Table 2:** Eczema outcomes in the first year of life.

	*n*	Total (*n* = 393), *n* (%)	Cesarean section born (*n* = 129), *n* (%)	Vaginally born (*n* = 264), *n* (%)
Presence of eczema				
UK diagnostic tool	360	110 (30.6)	35 (29.9)	75 (30.9)
Nurse diagnosis	366	60 (16.4)	24 (20.5)	36 (14.5)
Eczema severity based on POEM score (diagnosed with the UK diagnostic tool)				
At 3 months of age	29			
Clear		2 (6.9)	0 (0)	2 (9.5)
Mild		9 (31.0)	2 (25.0)	7 (33.3)
Moderate		13 (44.8)	5 (62.5)	8 (38.1)
Severe		5 (17.2)	1 (12.5)	4 (19.0)
At 6 months of age	64			
Clear		15 (23.4)	3 (15.0)	12 (27.3)
Mild		17 (26.6)	6 (30.0)	11 (25.0)
Moderate		30 (46.9)	11 (55.0)	19 (43.2)
Severe		2 (3.1)	0 (0)	2 (4.5)
At 9 months of age	70			
Clear		22 (31.4)	9 (42.9)	13 (26.5)
Mild		27 (38.6)	7 (33.3)	20 (40.8)
Moderate		19 (27.1)	5 (23.8)	14 (28.6)
Severe		2 (2.9)	0 (0)	2 (4.1)
At 12 months of age	68			
Clear		17 (25.0)	6 (28.6)	11 (23.4)
Mild		33 (48.5)	9 (42.9)	24 (51.1)
Moderate		17 (25)	5 (23.8)	12 (25.5)
Severe		1 (1.5)	1 (4.8)	0 (0)
Eczema severity at 12 months of age based on SCORAD score (nurse-diagnosed)				
	60			
Mild		48 (80.0)	18 (75)	30 (83.3)
Moderate		8 (13.3)	4 (16.7)	4 (11.1)
Severe		4 (6.7)	2 (8.3)	2 (5.6)
Age at onset of eczema (months)				
	110			
0–3		68 (61.8)	21 (60.0)	47 (62.7)
3–6		24 (21.8)	7 (20.0)	17 (22.7)
6–9		15 (13.6)	6 (17.1)	9 (12.0)
9–12		3 (2.7)	1 (2.9)	2 (2.7)
Topical steroid use				
During first 12 months of life	362	122 (33.7)	41 (34.7)	81 (33.2)

When evaluated by the UK diagnostic tool, eczema symptoms in participants started in 18.9% (68/360) at 0 to 3 months of age, in 6.7% (24/360) at 3 to 6 months of age, in 4.2% (15/360) at 6 to 9 months of age, and in 0.8% (3/360) at 9 to 12 months of age. The proportions of infants with different degrees of eczema severity based on POEM score were as follows: 6.9% (2/29) clear, 31% (9/29) mild, 44.8% (13/29) moderate, and 17.2% (5/29) severe at 3 months of age; 23.4% (15/64) clear, 26.6% (17/64) mild, 46.9% (30/64) moderate, and 3.1% (2/64) severe at 6 months of age; 31.4% (22/70) clear, 38.6% (27/70) mild, 27.1% (19/70) moderate, and 2.9% (2/70) severe at 9 months of age; and 25% (17/68) clear, 48.5% (33/68) mild, 25% (17/68) moderate, and 1.5% (1/68) severe at 12 months of age. The eczema severity at 12 months of age based on the SCORAD score was mild in 80% (48/60) of infants, moderate in 13.3% (8/60) of infants, and severe in 6.7% (4/60) of infants. During the first 12 months of life, steroids were administered topically to 33.7% (122/362) of infants (**Table 2**).

There was no association between eczema prevalence and clinical variables such as delivery mode sex and family history (**Supplementary File 2**, **Table 1**).

### Overall microbiome profile

On average, 7.3 million read pairs (range 107,000–41,000,000) were obtained per sample. Overall, 19 and 4 bacterial phyla, 212 and 48 families, 882 and 97 genera, and 2,806 and 286 species were identified by Kraken2/Bracken and MetaPhlAn 3, respectively.

Delivery mode, gestational age, formula milk, antibiotics during labor, and number of siblings had a strong effect on the bacterial composition at all taxonomic levels (PERMANOVA test, **Table 3**; **Supplementary File 2**, **Tables 2**-**4**). Birth weight had an effect on the composition of bacterial families, genera, and species (**Table 3**; **Supplementary File 2**, **Tables 3**, **4**). Concerning animal exposure, we did not find consistent results among the taxonomic levels: exposure to livestock at 12 months was significantly associated with bacterial composition at the phylum level only (**Supplementary File 2**, **Table 2**); exposure to pets (excluding cats and dogs) and livestock during pregnancy was associated with family, genus, and species composition (**Table 3**; **Supplementary File 2**, **Tables 3**, **4**). Childcare attendance was significantly associated with phylum composition, as investigated by MetaPhlAn 3 (**Supplementary File 2**, **Table 2**). No associations were found between microbiome composition and sex, ethnicity, antibiotic exposure during the first year of life, smoking during pregnancy, antibiotic use during the last trimester of pregnancy, presence of smokers in the household at 12 months of age, cat and dog exposure during pregnancy or at 12 months of age, familial factors (presence of atopic disease or allergies in any parent and/or sibling, or presence of atopic disease in both parents), or use of topical steroids during the first 12 months of life.

**Table 3:** Association between clinical variables and microbiome species composition.

Pipeline	Kraken					MetaPhlAn 3				
Factors	Samples (*n*)	Species (*n*)	Pseudo-F	p-value	R2	Samples (*n*)	Species (*n*)	Pseudo-F	p-value	R2
Infant										
Delivery mode	393	2,806	42.13	**0.0001**	0.0973	393	286	29.16	**0.0001**	0.0694
Sex	393	2,806	0.78	0.6651	0.0020	393	286	0.72	0.7781	0.0018
Ethnicity	393	2,806	1.18	0.2017	0.0090	393	286	1.28	0.1011	0.0098
Gestational age	393	2,806	7.41	**0.0001**	0.0186	393	286	5.73	**0.0001**	0.0144
Birth weight	393	2,806	3.65	**0.0004**	0.0093	393	286	2.97	**0.001**	0.0075
Formula milk before 7 days of life	393	2,806	5.18	**0.0001**	0.0131	393	286	4.51	**0.0001**	0.0114
Antibiotic exposure in first 12 months of life	393	2,806	1.29	0.1875	0.0033	393	286	1.49	0.0994	0.0038
Maternal										
Smoking during pregnancy	392	2,805	1.67	0.0638	0.0043	392	286	1.73	0.0411	0.0044
Antibiotic use during last trimester of pregnancy	393	2,806	1.06	0.337	0.0027	393	286	1.05	0.3741	0.0027
Antibiotics use during labor	391	2,805	2.76	**0.0053**	0.0071	391	286	3.07	**0.0012**	0.0078
Environmental										
Number of siblings	393	2,806	1.30	0.0816	0.0132	393	286	1.23	0.1105	0.0125
Childcare attendance in first 12 months of life	390	2,797	1.30	0.1829	0.0033	390	285	1.33	0.1593	0.0034
Animal exposure during pregnancy										
Pets in household	393	2,806	1.39	0.1455	0.0036	393	286	1.44	0.113	0.0037
Cats	393	2,806	0.74	0.7057	0.0019	393	286	0.92	0.5236	0.0024
Dogs	393	2,806	0.92	0.4783	0.0024	393	286	1.11	0.3109	0.0028
Other pets	393	2,806	1.90	**0.0397**	0.0048	393	286	1.59	0.0715	0.0040
Livestock	393	2,806	2.00	**0.0253**	0.0051	393	286	1.69	**0.0449**	0.0043
Animal exposure at 12 months of age										
Pets in household	378	2,786	1.03	0.3732	0.0027	378	284	1.08	0.3342	0.0029
Cats	378	2,786	0.56	0.92	0.0015	378	284	0.67	0.8425	0.0018
Dogs	378	2,786	0.87	0.5526	0.0023	378	284	1.20	0.237	0.0032
Other pets	378	2,786	0.84	0.5793	0.0022	378	284	1.01	0.4068	0.0027
Livestock	379	2,786	0.99	0.4153	0.0026	379	284	0.70	0.8104	0.0019
Familial										
Atopic disease, any parent and/or sibling	393	2,806	1.00	0.3986	0.0025	393	286	1.10	0.321	0.0028
Allergies, any parent and/or sibling	393	2,806	1.12	0.2974	0.0028	393	286	1.15	0.2891	0.0029
Atopic disease, both parents	393	2,806	0.67	0.8068	0.0017	393	286	0.82	0.6611	0.0021
Presence of eczema										
UK diagnostic tool	360	2,757	1.25	0.2057	0.0035	360	277	0.99	0.432	0.0028
Nurse diagnosis	366	2,765	1.17	0.2587	0.0032	366	281	0.94	0.5013	0.0026
Topical steroid use										
During first 12 months of life	362	2,715	1.06	0.3437	0.0029	362	277	1.13	0.3019	0.0031

Bold = p-values below 0.05.

No archaeal taxa were identified by MetaPhlAn 3. The only archaeal taxon detected by Kraken2\Bracken was the family of *Methanobacteriaceae* from the phylum *Euryarchaeota*. This family was detected in only two samples (read counts 12 and 28, respectively).

In total, 29 fungal species were identified by Kraken2\Bracken, belonging to 3 phylum, 21 families, and 21 genera. These species were identified in 68 (17.3%) samples and had read counts that did not allow further analyses (on average 0.03% of total non-human quality filtered reads). One fungal species (*Candida albicans*) was detected by MetaPhlAn 3 in three samples with a relative abundance between 0.02% and 0.29%. Because of the low abundance of archaea and fungi, the association between their composition in the intestinal microbiota and eczema outcomes could not be analyzed.

### Intestinal bacterial microbiome composition and the presence of eczema

**Figure 1:**
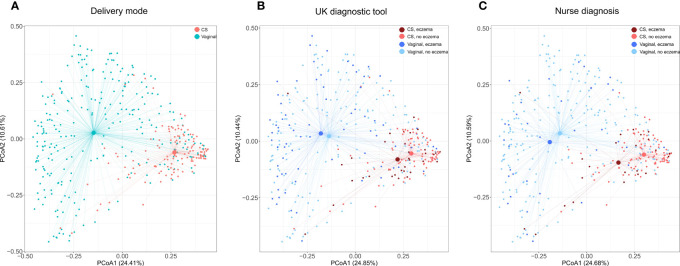
Species microbiome profiles according to delivery mode and eczema outcomes. Principal coordinate analyses (PCoAs) were performed on the relative abundance of 2,806 species detected in 393 infants with the Kraken2/Bracken pipeline. Axes indicate the percentage of total variance. Colors indicate delivery mode **(A)** and delivery mode and eczema outcomes **(B, C)**. For eczema outcomes, participants with missing values were excluded from the analysis and plotting; thus, **(B)** was computed on 91.6% (360/393) of samples and 98.3% (2,757/2,806) of species, and **(C)** on 93.1% (366/393) of samples and 98.5% (2,765/2,806) of species. The centroid indicates average microbiome species composition. Lines connect centroids to corresponding data points.

**Figure 2:**
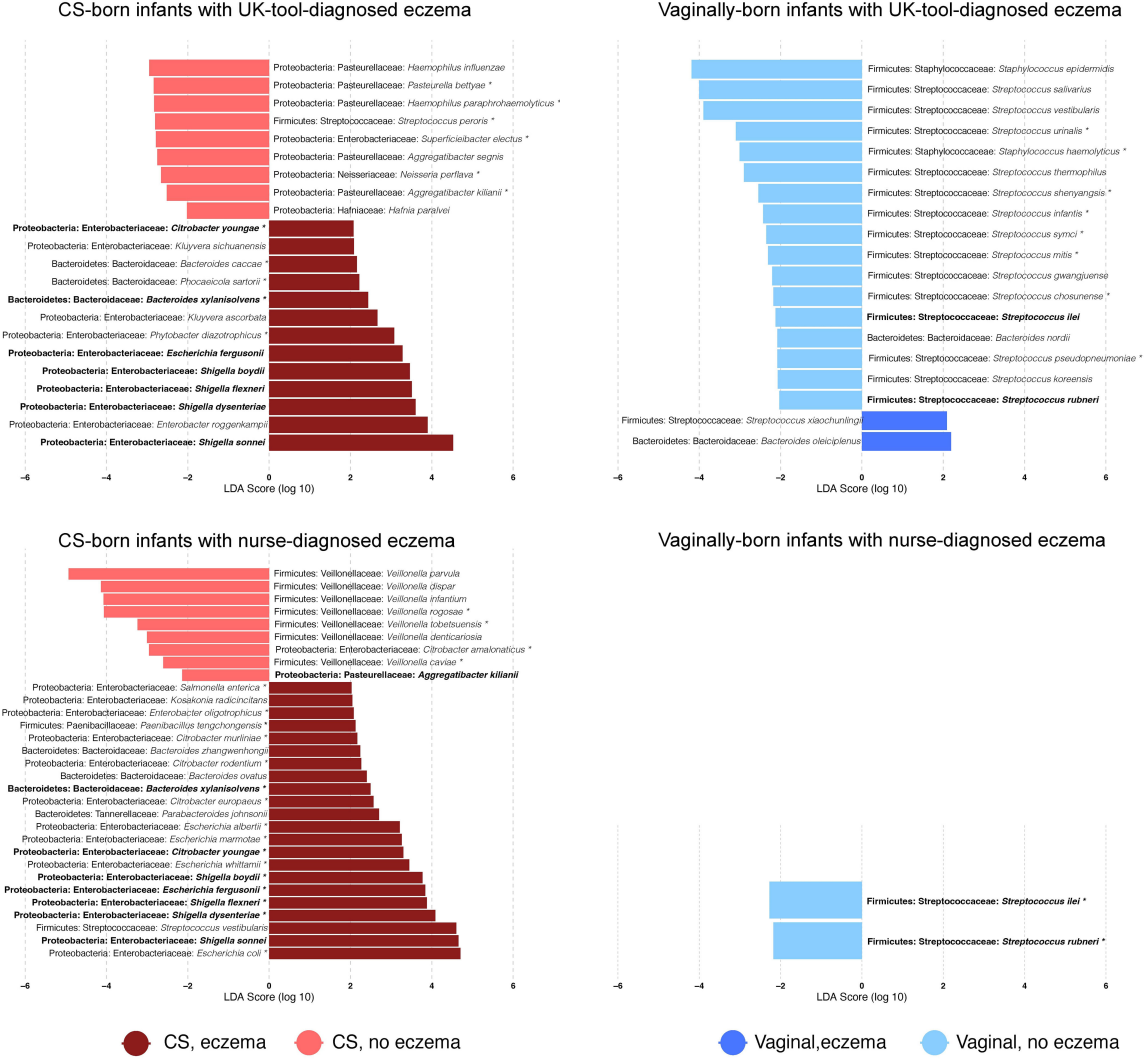
Differentially abundant bacterial species. Bar plots show the log10-transformed linear discriminant analysis (LDA) score for a given species obtained by LEfSe analysis according to the delivery mode and the presence of eczema diagnosed with a given tool. LDAs report which are the major species explaining the differences in microbiome species relative abundance between infants with and without eczema. Positive values of LDA correspond to species that are overrepresented in infants with eczema; negative values to those without eczema. * = species that were also detected as differentially abundant with DESeq2 (FDR-adjusted *p*-value < 0.05). Bold = species that were found to be differentially abundant by both eczema diagnosis tools in CS- or vaginally born infants. Values are also reported in **Supplementary File 3**.

As expected, in PCoA plots, the stool microbiome profiles of CS-born infants clustered further apart from those of vaginally born infants than the stool microbiome profiles of infants with eczema clustered from those of infants without eczema (**Figure 1**; **Supplementary File 2**, **Figures 1**-**3**). There was no difference in bacterial species diversity or richness between CS-born or vaginally born infants with or without eczema (**Supplementary File 2**, **Figure 4**). However, in CS-born infants with nurse-diagnosed eczema, the microbiome composition was significantly different at all taxonomic levels to that of CS-born infants without eczema (PERMANOVA test, *p*-values 0.0297, 0.0162, 0.0176, and 0.0125 for phylum, family, genus, and species, respectively; **Supplementary File 2**, **Table 5**).

Overall, the four most abundant phyla found in the intestinal microbiome were *Firmicutes*, *Proteobacteria*, *Bacteroidetes*, and *Actinobacteria*. No significant differences in the relative abundance of these phyla were detected in relation to eczema outcomes in either CS- or vaginally born infants (**Supplementary File 2**, **Figure 5**).

The 10 most abundant families were *Enterobacteriaceae*, *Bacteroidaceae*, *Bifidobacteriaceae*, *Veillonellaceae*, *Streptococcaceae*, *Staphylococcaceae*, *Pasteurellaceae*, *Tannerellaceae*, *Clostridiaceae*, and *Enterococcaceae*. Compared with CS-born infants without eczema, CS-born infants with eczema had a higher relative abundance of *Enterobacteriaceae* (*p*-values of 0.06 and 0.15 for the UK diagnostic tool and nurse diagnosis, respectively) and a lower relative abundance of *Veillonellaceae* (*Bacillota*) (*p*-values of 0.48 and 0.006 for the UK diagnostic tool and nurse diagnosis, respectively) (**Supplementary File 2**, **Figure 6**). Vaginally born infants with eczema had a lower relative abundance of *Streptococcaceae* than vaginally born infants without eczema (*p*-values 0.01 and 0.36 for the UK diagnostic tool and nurse diagnosis, respectively) (**Supplementary File 2**, **Figure 6**).

The 10 most abundant genera were *Bifidobacterium, Veillonella, Bacteroides, Escherichia, Streptococcus, Klebsiella, Shigella, Phocaeicola, Staphylococcus*, and *Parabacteroides*, and the 10 most abundant species were *Escherichia coli, Bifidobacterium longum, Veillonella parvula, Parabacteroides distasonis, Staphylococcus epidermidis, Haemophilus parainfluenzae, Streptococcus salivarius, Phocaeicola dorei, Bifidobacterium breve*, and *Shigella sonnei*. Genera and species, which were identified by Kraken2/Bracken and had >10 reads in at least one-quarter of the sample cohort, were kept for further analyses. After this filtering step, 12.6% (111/882) of genera and 11.6% (326/2806) of species were retained. CS-born infants with eczema had a higher relative abundance of the genera *Escherichia* and *Shigella* than CS-born infants without eczema (*p*-values 0.049 and 0.008, respectively, for nurse diagnosis) and a lower relative abundance of *Veillonella* (*p*-value 0.004 for nurse diagnosis) (**Supplementary File 2**, **Figure 7**). Although not among the 10 most abundant genera, *Enterobacter* and *Citrobacter* were also more abundant in CS-born infants with eczema than CS-born infants without eczema. Vaginally born infants with eczema had a higher abundance of *Bacteroides* and a lower abundance of *Streptococcus (p*-values 0.045 and 0.011, respectively, for the UK diagnostic tool) (**Figure 2**; **Supplementary File 2**, **Figures 7**, **8**).

The seven species *Bacteroides xylanisolvens, Citrobacter youngae, Shigella boydii, Escherichia fergusonii, Shigella flexneri, Shigella dysenteriae*, and *Shigella sonnei* were found to be discriminative for CS-born infants with eczema diagnosed by the UK diagnostic tool or nurse diagnosis (**Figure 2**), with a significantly higher abundance in infants with eczema. Seven *Veillonella* species, *V. parvula, V. dispar, V. infantium, V. rogosae, V. tobetsuensis, V. denticariosia*, and *V. caviae*, were found to be discriminative by LEfSe analyses, with a lower abundance in CS-born infants with nurse-diagnosed eczema (**Figure 2**; **Supplementary File 3**). Compared with vaginally born infants without eczema, *Streptococcus rubneri* and *Streptococcus ilei* were less abundant in vaginally born infants with both UK-diagnostic-tool- and nurse-diagnosed eczema.

### Metabolic pathways differentially abundant in the intestinal microbiome and the presence of eczema

Eighteen pathways were significantly overrepresented in CS-born infants with both UK-diagnostic-tool- and nurse-diagnosed eczema. These were linked to:

(1) amine and polyamine biosynthesis (superpathway of polyamine biosynthesis I);
(2) amino acid synthesis (L-ornithine biosynthesis II and superpathway of arginine and polyamine biosynthesis, L-methionine biosynthesis, L-homoserine and L-methionine biosynthesis, and S-adenosyl-L-methionine biosynthesis);
(3) antibiotic resistance (polymyxin resistance)
(4) carbohydrate degradation (L-rhamnose degradation I, chitin deacetylation, starch degradation III, and sucrose degradation II)
(5) cofactor, carrier, and vitamin biosynthesis (superpathway of pyridoxal 5-phosphate biosynthesis and salvage, and pyridoxal 5-phosphate biosynthesis I)
(6) fatty acid degradation (fatty acid beta-oxidation I and superpathway of glyoxylate cycle and fatty acid degradation); and
(7) Tricarboxylic acid (TCA cycle I, TCA cycle V, and superpathway of glyoxylate bypass). Pathways from amine and polyamine biosynthesis; amino acid synthesis; cofactor, carrier, and vitamin biosynthesis; and fatty acid degradation are present in *Escherichia coli* species according to the MetaCyc database. Genes involved in homolactic fermentation and biotin biosynthesis II were significantly overrepresented in CS-born infants without eczema when compared with CS-born infants with eczema, irrespective of diagnostic tool (**Supplementary File 2**, **Figure 9**).

Functional analyses of the vaginally born cohort did not result in overlapping findings between the UK diagnostic tool and nurse diagnosis.

### Intestinal phage composition and the presence of eczema

In total, viruses were detected in 379 infants (96.4%) and accounted for 0.2% (IQR 0.05%–0.48%) of quality-filtered non-human reads. To investigate phages, species belonging to the Caudovirales order were selected. These were the only detected viruses in 359 samples (91.3%). The most abundant detected phages were those infecting *Bacteroides*, *Enterococcus*, *Escherichia*, *Enterobacteria*, *Streptococcus*, and *Staphylococcus*. No significant differences were detected in the composition of phages according to eczema outcomes (**Supplementary File 2**, **Figure 10**).

## Discussion

In this study, in the first week of life, CS-born infants who subsequently developed eczema had a significantly different microbiome composition at all taxonomic levels from CS-born infants without eczema. Compared with the UK diagnostic tool, nurse diagnosis at 1 year of age is a more robust assessment as it captures infants with more severe and persistent eczema. This explains the larger differences in the composition of the intestinal microbiome observed between infants with and without nurse-diagnosed eczema compared with the differences observed using the UK diagnostic tool, as the latter also captures infants who might have only transitorily suffered from mild eczema.

Consistent with our findings, previous studies have also reported that infants who develop eczema have a higher relative abundance of *Enterobacteriaceae*, especially *Escherichia*, *Shigella*, and *Klebsiella*, in their intestinal microbiome (Penders et al., 2006; Penders et al., 2007; Hong et al., 2010; Ta et al., 2020). Furthermore, a higher relative abundance of *Enterobacteriaceae*, especially *Escherichia, Shigella, Enterobacter*, and *Citrobacter*, have been found in the intestinal microbiome of children who develop food allergies and asthma (Gosalbes et al., 2013; Azad et al., 2015; Zheng et al., 2016; Tanaka et al., 2017).

Similar to our findings, a lower relative abundance of *Veillonella* in the intestinal microbiome has been found to be associated with the development of atopic disease and food allergies in children (Arrieta et al., 2015; Chen et al., 2016; Tanaka et al., 2017). In contrast, one small study investigating the intestinal microbiome and the prevalence of eczema simultaneously at 1 year of age found a positive association between the relative abundance of *Veillonella* and eczema (Zheng et al., 2016). This could be explained by the intestinal microbiome being analyzed at 1 year of age rather than 1 week of age.

*Bacteroides*, especially *Bacteroides fragilis*, have previously been reported to be more abundant in the intestine of infants who develop eczema (Vael et al., 2008; Vael et al., 2011; Wang et al., 2016). We found this association only in infants who were vaginally born. This was likely to be because of the low abundance of *Bacteroides* in the CS-born infants, which is a common observation in infants born by CS (Zimmermann and Curtis, 2018). Immunoglobulin (Ig) A is produced in mucosal tissue, including at high concentrations in the intestine. IgA promotes intestinal barrier function and is involved in the maintenance of host–commensal mutualism (Fagarasan et al., 2010). The binding of IgA to *Bacteroides thetaiotaomicron* inhibits the innate immune response (Peterson et al., 2007). *Bacteroides* are the main producer of the short-chain fatty acid propionate (Louis and Flint, 2017) and have been associated with both increased and decreased inflammation of the intestine (Hertli and Zimmermann, 2022). Propionate increases glucose-6-phosphatase activity in the jejunum, which induces gluconeogenesis (Kimura et al., 2011; De Vadder et al., 2014; Chenard et al., 2020). Increased glucose levels in stool samples have previously been found in infants who develop eczema (Ta et al., 2020). However, in the study that found increased glucose levels, infants who developed eczema were reported to have delayed colonization by *B. fragilis.* It has been hypothesized that the increased glucose levels result from a depletion of bacterial groups with the ability to derive butyrate and propionate through direct or indirect pathways, which results in reduced glycolysis (Ta et al., 2020).

A lower relative abundance of *Streptococcus* in the intestinal microbiome has previously been associated with the development of eczema in children (Zheng et al., 2016; Yu et al., 2021). In our study, we found a lower relative abundance of *Streptococcus* in both CS- and vaginally born infants with eczema; however, the difference was statistically significant only in the latter. This could be because a higher proportion of infants born by CS received formula milk, which has been associated with a lower abundance of *Streptococcus* (Zimmermann and Curtis, 2018).

Previous studies have reported an association between a lower relative abundance of *Bifidobacterium* and the development of eczema (Watanabe et al., 2003; Murray et al., 2005; Mah et al., 2006; Hong et al., 2010; Penders et al., 2010). Although *Bifidobacterium* was among the most abundant genera identified in our study, we did not observe such an association. Similarly, *Clostridioides difficile* has previously been found to be more abundant in the first month of life in infants who develop eczema (Penders et al., 2007; van Nimwegen et al., 2011), an association we also did not find in our study. In mice, *Clostridia*-related bacteria called segmented filamentous bacteria are reported to promote interleukin (IL)-6 and IL-26 production by dendritic cells, which leads to the induction of T helper (Th) 17 cell differentiation (Ivanov et al., 2009). Th17 cells produce pro-inflammatory cytokines such as IL-17A, IL-17F, and IL-22, which have been associated with the development of autoimmune disease (Littman and Rudensky, 2010). *Clostridium* spp. cluster IV and XIVa strains, as well as *B. fragilis*, have been associated with an increase in IL-10-producing T regulatory (Treg) cells (Atarashi et al., 2011; Round et al., 2011). Treg cells accumulate in the intestine and play a critical role in the maintenance of immune homeostasis and the development of autoimmunity and allergies (Turner et al., 2020).

A higher relative abundance of *Lactobacillus paracasei* in the early intestinal microbiome has been found to reduce the risk of developing eczema (Penders et al., 2010). Administered as a probiotic, *Lactobacillus rhamnosus*, which belongs to the same taxonomic group as *L. paracasei*, halves the risk of developing eczema (Wickens et al., 2008). In *in vitro* studies, exopolysaccharides from *L. rhamnosus* have been shown to stimulate TNF, IL-6, and IL-12 secretion from human peripheral blood mononuclear cells (Chabota et al., 2001).

In our study, a lower abundance of genes involved in lactic fermentation was found in CS-born infants with eczema. *Veillonella* metabolize lactate, the final product of lactic fermentation, into the short-chain fatty acids acetate and propionate (Ng and Hamilton, 1973). These metabolites have anti-inflammatory activities and can help to maintain the function of the intestinal barrier (Nurmi et al., 2005; Tedelind et al., 2007; Suzuki et al., 2008). It remains unclear whether lactate has pro- or anti-inflammatory properties (Manosalva et al., 2022). It is also not yet known whether a lower abundance of *Veillonella* is associated with higher levels of lactate and whether this is associated with an increase in intestinal inflammation. Other bacteria that can ferment lactate, such as *Lactobacillus*, *Streptococcus*, and *Bifidobacterium*, have also been found to be less abundant in infants who develop eczema (Watanabe et al., 2003; Murray et al., 2005; Mah et al., 2006; Hong et al., 2010; Penders et al., 2010; Zheng et al., 2016; Yu et al., 2021).

Although it has been reported that being born by CS and receiving formula milk increases the risk of developing eczema (Kull et al., 2005; Yu et al., 2015), the evidence for this is not robust. In fact, recent studies and meta-analyses report that infants born by CS do not have an increased risk for developing eczema (Renz-Polster et al., 2005; Bager et al., 2008; Polos and Fletcher, 2019). Similarly, a protective effect of breastfeeding against developing eczema is not certain (Elbert et al., 2017; Wang et al., 2017; Bigman, 2020). In line with this, there is only a partial overlap between the differences found in the intestinal microbiome after being born by CS or receiving formula milk and the differences in the composition of the intestinal microbiome associated with the development of eczema. Infants born by CS are known to have a higher relative abundance of *Clostridioides* and *Enterobacteriaceae* other than *Escherichia* (e.g., *Citrobacter* and *Enterobacter*) in their intestinal microbiome, while the relative abundance of *Escherichia* has been reported to be lower (Zimmermann and Curtis, 2018). Furthermore, infants born by CS have a lower relative abundance of *Bacteroides* and *Bifidobacterium*, while the findings on the abundance of *Streptococcus* remain contradictory (Zimmermann and Curtis, 2018). A higher relative abundance of *Veillonella* has been found in infants born by CS (Hesla et al., 2014). Similarly, breastfed infants have been reported to have a higher relative abundance of *Bifidobacterium* and a lower relative abundance of *Bacteroides* and *Clostridioides*, while the findings for *Streptococcus* and *Veillonella* are contradictory (Zimmermann and Curtis, 2018).

Although preterm-born infants are known to have a higher relative abundance of *Enterobacteriaceae* (especially *Escherichia, Enterobacter*, and *Citrobacter*) and *Bacteroides*, as well as a lower relative abundance of *Bifidobacterium*, in their intestinal microbiome (Zimmermann and Curtis, 2018), they have not been found to develop eczema more frequently than term-born infants (Gerner et al., 2022). However, in addition to the findings above, preterm-born infants have also been found to have a higher relative abundance of *Streptococcus* and *Veillonella* (Zimmermann and Curtis, 2018), which might protect them from eczema. Infants exposed to antibiotics have been reported to have a significantly higher risk of developing eczema and other allergic diseases (Duong et al., 2022). Although infants exposed to antibiotics have been reported to have a lower relative abundance of *Bifidobacterium* and a higher relative abundance of *Enterobacteriaceae* in their intestinal microbiome, they have also been found to have a lower relative abundance of *Bacteroides*, while findings for *Clostridioides* remain contradictory (Zimmermann and Curtis, 2018). However, the changes in the intestinal microbiome induced by antibiotics are influenced by many other factors, such as the condition leading to the antibiotic use, underlying disease, hospital admission, and, even more importantly, the dose, duration, administration route, and spectrum of the antibiotic (Zimmermann and Curtis, 2019). Exposure to pets has been associated with a higher relative abundance of *Veillonella* (Azad et al., 2013), which might partially explain why children exposed to pets are less prone to eczema (Epstein et al., 2011). However, again, the association between pet exposure and a decreased risk of eczema is not clear (Langan et al., 2007).

In conclusion, our study of a large cohort of infants, using shotgun metagenomic sequencing and independent bioinformatic and statistical pipelines, found that the bacterial composition of the intestinal microbiome at 1 week of age is associated with the subsequent presence of eczema in the first year of life. Our study also identified putative genera (*Escherichia, Shigella, Enterobacter, Citrobacter*, and *Bacteroides*) that might play a role in the development of eczema and others (*Streptococcus* and *Veillonella*) that seem to be protective.

The limitations of our study include the fact that the bioinformatics analyses identified taxa by mapping reads to known reference genomes without using *de novo* assembly. Furthermore, reads were mapped individually in each sample. In future, new approaches based on assembling metagenomes by pooling reads from all samples might improve the analyses of the composition of the microbiome, while also enabling the recovery of the genome of community-shared strains that are not yet discovered (Uritskiy et al., 2018; Churcheward et al., 2022; Krakau et al., 2022). The DNA extraction protocol used was not specifically designed to target viral particles, which means that the abundance of bacteriophages might have been underestimated. Also, many intestinal phages are yet to be characterized (Sutton and Hill, 2019).

Differences between the findings in our study and other studies investigating the association between the intestinal microbiome and the risk of developing eczema might be explained by (1) differences in the investigated time points, i.e., 1 week of age might be too early to detect significant differences in the abundance of *Lactobacillus*, *Bifidobacterium*, and *Clostridioides*, which are more prevalent later in infancy; (2) differences in the definition of eczema outcomes; (3) differences in the methodologies used to investigate the microbiome; and (4) smaller cohort sizes.

Future studies should include multiple sampling time points to investigate the trajectory of the intestinal microbiome that is associated with the development of eczema and atopic disease in general. Furthermore, the exact mechanistic roles of individual bacterial species and the role of the interaction between these species in atopic diseases should be addressed. Findings from these studies will form the basis for evidence-based interventions to modulate the intestinal microbiome in infants and children at risk of eczema and atopic disease, such as modifying breast milk and intestinal microbiota with directed prebiotics and probiotics, and the use of bacteriophages.

## Data availability statement

FASTQ files containing forward and reverse quality-filtered non-human reads were deposited in the European Nucleotide Archive (https://www.ebi.ac.uk/ena) under the project PRJEB55714. Codes used for the preprocessing of sequencing reads (quality-filtering, removal of human DNA, etc.) are deposited at GitHub: https://github.com/miraclelabunifr/Eczema.

## Ethics statement

The studies involving human participants were reviewed and approved by the Royal Children’s Hospital Human Research Ethics Committee HREC, authorisation (53543). Written informed consent to participate in this study was provided by the participants’ legal guardians/next of kin.

## Author contributions

PZ conceptualized and supervised the study. NC is the chief principal investigator of the MIS BAIR trial. WJ performed the sequencing. SL, PZ, LP and NM helped with data cleaning. SL, OC, LP, PZ, and LF analyzed the data. SL and PZ wrote the initial draft of the manuscript. SL, OC, LP, LF, NC, and PZ revised the manuscript. All authors contributed to the article and approved the submitted version.

## Supplementary material

Supplementary File 1 R code.

Supplementary File 2 Supplementary figures and tables.

Supplementary File 3 Excel tables containing results of LEfSe and DESeq2 analyses.
