# Supplementary material for: The association between the composition of the early-life intestinal microbiome and eczema in the first year of life
Source: Front Microbiomes. 2023 Mar 16;2:1147082. doi: 10.3389/frmbi.2023.1147082 (PMC12993559; doi:10.3389/frmbi.2023.1147082)
Supplement: Supplementary File 1 — R code. [file DataSheet_1.zip › Supplementary File 2.html]

Supplementary\_materials\_software\_misbair\_eczema


# Supplementary\_materials\_software\_misbair\_eczema

#### 2022-12-13

## Load R libraries, set working directory, read tables

For simplicity, we give information for species analyses. The coding
for the other taxonomic levels is the same.

```
library(dplyr) # data frame manipulation and pipe operator
library(phyloseq) # build phyloseq object
library(tidyr)
library(tidyverse) 
library(ggplot2)
library(ggpubr) #for ggboxplot function
library(microbiomeMarker) # lefse analysis function
library(microViz) # filtering phyloseq object
library(cowplot) # plot_grid() function which is used to merge ggplot objects
library(gridExtra) # grid.arrange() another function used to merge ggplot objects 
library(grid) # text_grob() function used to write colored text
library(gridtext)
library(vegan)
library(DESeq2)
library(openxlsx)
library(readxl)

#setwd
#setwd("/data/")

#read table from Kraken2/Bracken
species_bracken = read.delim("bac_vir_fung_counts_species_bracken.csv", header =TRUE, sep =",")
#rearrange table and correct sampleID so that it matches the metadata
colnames(species_bracken)=  gsub("Counts_no_human_", "", colnames(species_bracken))
colnames(species_bracken) = gsub("\\.", "-", colnames(species_bracken))

#keep only bacteria
species_bracken = species_bracken[grep("d__Bacteria", species_bracken$Taxonomy),]

#label rownames with species name and taxID
rownames(species_bracken) = paste(as.character(species_bracken$Species),  as.character(species_bracken$TaxID), sep ="_&_") 
#remove first three columns containg character values
species_bracken = species_bracken[, -which(colnames(species_bracken) %in% c("Species", "TaxID", "Taxonomy"))]

#convert NA counts in 0
species_bracken[is.na(species_bracken)] = 0

#read table from Metaphlan
species_metaphlan = read.table("merged_abundance_table.txt", sep="\t", skip =1, header = TRUE)

#rearrange table and correct sampleID so that it matches the metadata
colnames(species_metaphlan) = gsub("results_no_human_","",  colnames(species_metaphlan))
colnames(species_metaphlan)[-c(1:2)] = gsub('.{1}$', '',colnames(species_metaphlan)[-c(1:2)] )
colnames(species_metaphlan)[which(colnames(species_metaphlan) == "3778D6.2_S30")] = "3778D6-2_S30"

#put species and taxonomy as row.names 
rownames(species_metaphlan) = as.character(species_metaphlan$clade_name)
#remove first two columns (character values)
species_metaphlan = species_metaphlan[,-c(1:2)]

#filter data only for species
species_metaphlan <- species_metaphlan[grep("k__Bacteria", rownames(species_metaphlan)),]
species_metaphlan <- species_metaphlan[grep("\\|s__", rownames(species_metaphlan)),]

#read metadata 

metadata = read.xlsx("metadata.xlsx")
metadata = as.data.frame(metadata)
 
metadata$SampleID = as.character(metadata$SampleID )
metadata$select_correct = as.character(metadata$select_correct)

mysamples = metadata$SampleID[metadata$select_correct=="yes"]

species_bracken = species_bracken[,colnames( species_bracken) %in% mysamples]
species_metaphlan = species_metaphlan[,colnames(species_metaphlan) %in% mysamples]

#compute relative abundance
species_bracken_prop = apply(species_bracken,2,function(x) (x/sum(x))*100)
```

How the bracken read count table looks like:

```
head(species_bracken[,1:10])
```

```
##                                      3126D7_S1 3159D7_S1 3182D5_S2 3189D7_S1
## Streptococcus salivarius_&_1304            207    512224    121903      9671
## Staphylococcus epidermidis_&_1282       853923      1821    380758     25860
## Staphylococcus caledonicus_&_2741333       441         0        53         0
## Streptococcus parasanguinis_&_1318        5008     21486       424      7434
## Streptococcus vestibularis_&_1343          571     63871     24415      2428
## Streptococcus lactarius_&_684066          1009      3265       590       865
##                                      3200D6_S3 3201D7_S2 3202D7_S4 3222D7_S9
## Streptococcus salivarius_&_1304            630     75374         0    175811
## Staphylococcus epidermidis_&_1282        65684    419456       249     70626
## Staphylococcus caledonicus_&_2741333        20        18         0        17
## Streptococcus parasanguinis_&_1318       55834       120       246      4818
## Streptococcus vestibularis_&_1343          744      5470         0   3109070
## Streptococcus lactarius_&_684066          6020        94        26       677
##                                      3233D7_S1 3234D7_S5
## Streptococcus salivarius_&_1304         167499    105496
## Staphylococcus epidermidis_&_1282        44655     67258
## Staphylococcus caledonicus_&_2741333        22         0
## Streptococcus parasanguinis_&_1318        2424     98588
## Streptococcus vestibularis_&_1343        32571    896344
## Streptococcus lactarius_&_684066          1038     11639
```

## Function used to compute principal coordinate analysis (PCoA)

For one variable:

```
mypcoa_1var = function(x, variable, metadata){
  require(vegan)
  require(ggplot2)
  
  #convert metadata variable in character
  metadata[, variable] = as.character( metadata[, variable])
  
  # built metadata table for PCOA:
  
  x.env = data.frame(SampleID= colnames(x))
  x.env$SampleID= as.character(x.env$SampleID)
  x.env$variable= metadata[,variable][match(x.env$SampleID, metadata$SampleID)]
  x.env$variable[is.na(x.env$variable)] = "none"
  x.env$variable[x.env$variable == "NA"] = "none"
  x.env$variable[x.env$variable == ""] = "none"
  
  #remove NA values in metadata
  n.samples.before = ncol(x)
  sam_to_remove=  x.env$SampleID[ x.env$variable == "none"]

  
  #remove samples with no information in variables
  if(identical(sam_to_remove, character(0))){
    x=x
    x.env = x.env
  
  }else{
    x.env = x.env[!x.env$SampleID %in% sam_to_remove,] 
    x = x[, -which(colnames(x) %in% sam_to_remove )]
  }
  
  
  #remove species that had 0 counts over the selected samples
  x = x[rowSums(x)>0,]
  
  #report n of species and no of samples after selection
  n.samples = ncol(x)
  n.species = nrow(x)
  
  #are datasets aligned?
  if(all(x.env$SampleID == colnames(x))){
    print("metadata and read counts table columns are aligned")
  }else{stop("metadata and read counts table columns are NOT aligned")}
 
  #transf. sqrt 
  y= apply(x, 2, function(x) sqrt(x))
  
  ## Bray-Curtis distances between samples
  dis <- vegdist(t(y), dist ="bray")
  
  ## create groups
  groups <- as.factor(x.env$variable)
 
  ## Calculate multivariate dispersions
  mod <- betadisper(dis, group = groups)
  mydf = as.data.frame(mod$vectors)
  
  #compute %var axes
  var_values <- round(100*mod$eig / sum(mod$eig), 2)
  
  mydf$variable = as.character(x.env$variable[match(rownames(mydf),x.env$SampleID)])
  #add centroids
  centroids = as.data.frame(mod$centroids)
  mydf$PCoA1_centr = centroids$PCoA1[match(mydf$variable, rownames(centroids))]
  mydf$PCoA2_centr = centroids$PCoA2[match(mydf$variable, rownames(centroids))]
  
  mytitle = paste(variable,  n.samples , "out of",  n.samples.before, "on", n.species, "species", sep =" ")
      
  
  pcoa_plot = ggplot(mydf, aes(x = PCoA1, y = PCoA2)) + geom_point(aes(color =variable, alpha = 0.5))+
    ggtitle(mytitle)+
    geom_segment(aes(x=PCoA1_centr, y=PCoA2_centr, xend=PCoA1, yend=PCoA2, alpha = 0.3,color =variable))+
    geom_point(aes(x=PCoA1_centr,y=PCoA2_centr, color =variable),size=5)+
    labs(x = paste("PCoA1 (",round(var_values[1],2), "%)", sep =""), 
         y = paste("PCoA2 (",round(var_values[2],2), "%)", sep =""))
  return(pcoa_plot)
}
```

By combining two variable:

```
mypcoa_2var = function(x, variables, metadata){
  require(vegan)
  require(ggplot2)

  #convert metadata variable in character
  for(i in 1:length(variables)){
    metadata[, variables[i]] = as.character( metadata[, variables[i]])
  }

  # built metadata table for PCOA:

  x.env = data.frame(SampleID= colnames(x))
  x.env$SampleID= as.character(x.env$SampleID)

  for(i in 1:length(variables)){
  x.env$variable= metadata[,variables[i]][match(x.env$SampleID, metadata$SampleID)]
  x.env$variable[is.na(x.env$variable)] = "none"
  x.env$variable[x.env$variable == "NA"] = "none"
  x.env$variable[x.env$variable == ""] = "none"
  colnames(x.env)[colnames(x.env) == "variable"] = paste("variable", i, sep =".")
  }

  x.env$mergev = paste( x.env$variable.1,x.env$variable.2, sep ="_" )

  #remove NA values in metadata
  n.samples.before = ncol(x)
  sam_to_remove=  x.env$SampleID[ grep("none",x.env$mergev)]

  #remove samples with no information in variables
  x.env = x.env[!x.env$SampleID %in% sam_to_remove,]
  x = x[, -which(colnames(x) %in% sam_to_remove )]

  #remove species that had 0 counts over the selected samples
  x = x[rowSums(x)>0,]

  #report n of species and no of samples after selection
  n.samples = ncol(x)
  n.species = nrow(x)

  #are datasets aligned?
  if(all(x.env$SampleID == colnames(x))){
    print("metadata and read counts table columns are aligned")
  }else{stop("metadata and read counts table columns are NOT aligned")}

  #transf. sqrt
  y= apply(x, 2, function(x) sqrt(x))

  ## Bray-Curtis distances between samples
  dis <- vegdist(t(y), dist ="bray")

  ## create groups
  groups <- as.factor(x.env$mergev)

  ## Calculate multivariate dispersions
  mod <- betadisper(dis, group = groups)
  mydf = as.data.frame(mod$vectors)

  #compute %var axes
  var_values <- round(100*mod$eig / sum(mod$eig), 2)

  mydf$variable = as.character(x.env$mergev[match(rownames(mydf),x.env$SampleID)])
  #add centroids
  centroids = as.data.frame(mod$centroids)
  mydf$PCoA1_centr = centroids$PCoA1[match(mydf$variable, rownames(centroids))]
  mydf$PCoA2_centr = centroids$PCoA2[match(mydf$variable, rownames(centroids))]


  mytitle = paste(paste(variables, collapse ="_"),  n.samples , "out of",  n.samples.before, "on", n.species, "species", sep =" ")

  pcoa_plot = ggplot(mydf, aes(x = PCoA1, y = PCoA2)) + geom_point(aes(color =variable, alpha = 0.5))+
    ggtitle(mytitle)+
    geom_segment(aes(x=PCoA1_centr, y=PCoA2_centr, xend=PCoA1, yend=PCoA2, alpha = 0.3,color =variable))+
    geom_point(aes(x=PCoA1_centr,y=PCoA2_centr, color =variable),size=5)+
    labs(x = paste("PCoA1 (",round(var_values[1],2), "%)", sep =""),
         y = paste("PCoA2 (",round(var_values[2],2), "%)", sep =""))

  return(pcoa_plot)
}
```

Here an example: PCoA for species detected with Kraken2/Bracken and
according to delivery mode.

```
## [1] "metadata and read counts table columns are aligned"
```

PCoA for species detected with Kraken/Bracken and according to
delivery mode and nurse diagnosis.

```
## [1] "metadata and read counts table columns are aligned"
```

## Function used to run PERMANOVA tests with adonis2

One variable:

```
run_permanova_global = function(x, metadata, variable,mycontinous_variables){
  require(vegan)

  #PERMANOVA for continous variables:
  
  if(variable %in% mycontinous_variables){
    # built metadata
    
    x.env = data.frame(SampleID= colnames(x))
    x.env$SampleID= as.character(x.env$SampleID)
    x.env$variable= metadata[,variable][match(x.env$SampleID, metadata$SampleID)]
    
    #remove na values from the x and env
    n.samples.before = ncol(x)  
    
    if(any(is.na(x.env$variable))){
      
      #which samples contain NA values?
      
      sample_to_remove = x.env$SampleID[is.na(x.env$variable)]
      
      x.env = x.env[!x.env$SampleID %in% sample_to_remove,]
      x = x[, colnames(x) %in% x.env$SampleID]
      
    }else{
      x.env = x.env
      x = x
    }
    
    #remove species that had 0 counts over the selected samples
    x = x[rowSums(x)>0,]
    
    #report n of species and no of samples after selection
    n.samples = ncol(x)
    n.species = nrow(x)
    
    #are datasets aligned?
    if(all(x.env$SampleID == colnames(x))){
      print("metadata and read counts table columns are aligned")
    }else{stop("metadata and read counts table columns are NOT aligned")}
    
    #variable
    
    mypermanova = adonis2(sqrt(t(x))~ variable, data = x.env , method ="bray", permutations =9999)
    
    #collect infos
    mydf = data.frame(n.samples.before = n.samples.before,n.samples.after = n.samples, 
                      n.species = n.species,pseudoF =  mypermanova$F[1],  p.value= mypermanova[,"Pr(>F)"][1],
                      r.square = mypermanova$R2[1], by.arg = "terms", variable = variable)
    
    return(list(mydf, mypermanova))
    
    
    #PERMANOVA one variable:
  } else if(!variable %in% mycontinous_variables){
    
    #convert metadata variable in character
    metadata[, variable] = as.character( metadata[, variable])
    
    # built metadata
    
    x.env = data.frame(SampleID= colnames(x))
    x.env$SampleID= as.character(x.env$SampleID)
    x.env$variable= metadata[,variable][match(x.env$SampleID, metadata$SampleID)]
    x.env$variable[is.na(x.env$variable)] = "none"
    x.env$variable[x.env$variable == "NA"] = "none"
    x.env$variable[x.env$variable == ""] = "none"
    x.env$variable[x.env$variable == "Missing"] = "none"
    x.env$variable[x.env$variable == "not_known"] = "none"
    x.env$variable[x.env$variable == ".a"] = "none"
    
    #remove NA values in metadata
    #report no of samples before the selection
    n.samples.before = ncol(x) 
    sam_to_remove=  x.env$SampleID[ x.env$variable == "none"] 
    
    if(identical(sam_to_remove, character(0))){
      
      x.env = x.env
      x = x
      
    }else{
      #remove samples with no information for the tested variable
      x.env = x.env[!x.env$SampleID %in% sam_to_remove,] 
      x = x[, -which(colnames(x) %in% sam_to_remove )]
    }
    
    #remove species that had 0 counts over the selected samples
    x = x[rowSums(x)>0,]
    
    #report n of species and no of samples after selection
    n.samples = ncol(x)
    n.species = nrow(x)
    
    #are datasets aligned?
    if(all(x.env$SampleID == colnames(x))){
      print("metadata and read counts table columns are aligned")
    }else{stop("metadata and read counts table columns are NOT aligned")}
    
    #variable
    
    mypermanova = adonis2(sqrt(t(x))~ variable, data = x.env , method ="bray", permutations =9999)
    
    #collect infos
    mydf = data.frame(n.samples.before = n.samples.before,n.samples.after = n.samples, 
                      n.species = n.species,pseudoF =  mypermanova$F[1],  p.value= mypermanova[,"Pr(>F)"][1],
                      r.square = mypermanova$R2[1], by.arg = "terms", variable = variable)
    
    return(list(mydf, mypermanova))
    
  }
}
```

By combining two variables:

```
#re-arrage table to give to adonis2 for pairwise comparison (e.g. CS-born, eczema vs CS-born no eczema)

rearr_tables = function(x, metadata, variable, variable2 = NULL){
  
  if(is.null(variable2)){
    
    #convert metadata variable in character
    metadata[, variable] = as.character( metadata[, variable])
    
    # built metadata
    
    x.env = data.frame(SampleID= colnames(x))
    x.env$SampleID= as.character(x.env$SampleID)
    x.env$variable= metadata[,variable][match(x.env$SampleID, metadata$SampleID)]
    x.env$variable[is.na(x.env$variable)] = "none"
    x.env$variable[x.env$variable == "NA"] = "none"
    x.env$variable[x.env$variable == ""] = "none"
    
    #remove NA values in metadata
    #report no of samples before the selection
    n.samples.before = ncol(x) 
    sam_to_remove=  x.env$SampleID[ x.env$variable == "none"] 
    
    #remove samples with no information for the tested variable
    if(identical(sam_to_remove, character(0))){
      
      x.env = x.env
      x = x
      
    }else{
      #remove samples with no information for the tested variable
      x.env = x.env[!x.env$SampleID %in% sam_to_remove,] 
      x = x[, -which(colnames(x) %in% sam_to_remove )]
    }
    
    #remove species that had 0 counts over the selected samples
    x = x[rowSums(x)>0,]
    
    #report n of species and no of samples after selection
    n.samples = ncol(x)
    n.species = nrow(x)
    
    #are datasets aligned?
    if(all(x.env$SampleID == colnames(x))){
      print("metadata and read counts table columns are aligned")
    }else{stop("metadata and read counts table columns are NOT aligned")}
    
    #traspose columns with sample names in rows and taxa names in columns
    x1 = t(x)
    
    if(all(x.env$SampleID == rownames(x1))){
      print("metadata and read counts table (t) columns are aligned")
    }else{stop("metadata and read counts table columns are NOT aligned")}
    
    
  }else{
    #bind variables
    variables = c(variable, variable2)
    
    #convert metadata variable in character
    for(i in 1:length(variables)){
      metadata[, variables[i]] = as.character( metadata[, variables[i]])
    }
    
    # built metadata table
    
    x.env = data.frame(SampleID= colnames(x))
    x.env$SampleID= as.character(x.env$SampleID)
    
    for(i in 1:length(variables)){
      x.env$variable= metadata[,variables[i]][match(x.env$SampleID, metadata$SampleID)]
      x.env$variable[is.na(x.env$variable)] = "none"
      x.env$variable[x.env$variable == "NA"] = "none"
      x.env$variable[x.env$variable == ""] = "none"
      colnames(x.env)[colnames(x.env) == "variable"] = paste("variable", i, sep =".") 
    }
    
    x.env$mergev = paste( x.env$variable.1,x.env$variable.2, sep ="_" )
    
    #remove NA values in metadata
    n.samples.before = ncol(x)
    sam_to_remove=  x.env$SampleID[ grep("none",x.env$mergev)] 
    
    #remove samples with no information in variables
    x.env = x.env[!x.env$SampleID %in% sam_to_remove,] 
    x = x[, -which(colnames(x) %in% sam_to_remove )]
    
    #remove species that had 0 counts over the selected samples
    x = x[rowSums(x)>0,]
    
    #report n of species and no of samples after selection
    n.samples = ncol(x)
    n.species = nrow(x)
    
    #are datasets aligned?
    if(all(x.env$SampleID == colnames(x))){
      print("metadata and read counts table columns are aligned")
    }else{stop("metadata and read counts table columns are NOT aligned")}
    
    #traspose columns with sample names in rows and taxa names in columns
    x1 = t(x)
    
    if(all(x.env$SampleID == rownames(x1))){
      print("metadata and read counts table (t) columns are aligned")
    }else{stop("metadata and read counts table columns are NOT aligned")}
    
    
  }
  
  return(list(x1, x.env))
}

#########################################################################################

#pairwise PERMANOVA
#adonis2

pairwise.adonis2 <- function(x,x.env, variable, sim.method = 'bray', p.adjust.m ='bonferroni', perms = 9999){
  require(vegan)
  #create comparison
  co = combn(unique(as.character(x.env[,variable])),2)
  pairs = c()
  F.Model =c()
  R2 = c()
  p.value = c()
  n.samples = c()
  n.species = c()
  for(elem in 1:ncol(co)){
    
    x1 = x[x.env[,variable] %in% c(co[1,elem],co[2,elem]),] 
    
    x.env1 = x.env[x.env[,variable] %in% c(co[1,elem],co[2,elem]),]
    colnames(x.env1)[colnames(x.env1) == variable] = "variable"
    
    ad = adonis2(x1 ~variable, data =x.env1 , method =sim.method, permutations = perms)
    ad$aov.tab = as.data.frame(ad[1:5])
    
    pairs = c(pairs,paste(co[1,elem],'vs',co[2,elem]));
    F.Model =c(F.Model,ad$aov.tab[1,4]);
    R2 = c(R2,ad$aov.tab[1,3]);
    p.value = c(p.value,ad$aov.tab[1,5])
    n.samples = c(n.samples, nrow(x1))
    n.species = c(n.species, ncol(x1))
  }
  p.adjusted = p.adjust(p.value,method=p.adjust.m)
  sig = c(rep('',length(p.adjusted)))
  sig[p.adjusted <= 0.05] <-'.'
  sig[p.adjusted <= 0.01] <-'*'
  sig[p.adjusted <= 0.001] <-'**'
  sig[p.adjusted <= 0.0001] <-'***'
  
  pairw.res = data.frame(pairs,F.Model,R2,p.value,p.adjusted,sig, n.samples, n.species)
  print("Signif. codes:  0 ‘***’ 0.001 ‘**’ 0.01 ‘*’ 0.05 ‘.’ 0.1 ‘ ’ 1")
  return(pairw.res)
  
}
```

## Differentially abundant species analyses

Selection of species detected with Kraken2/Bracken for DESeq2 and
LEfSe analyses.

```
#select species based on counts
#same criteria were used for genera
keep_species <- rowSums(species_bracken >= 10) >= 100
```

## DESeq2

Function used to perform DESeq2 analyses

```
run_deseq2 = function(x, metadata, variables, delivery){

#add 1 to all species to avoid zeros (pseudo-counts)
x = x+1

#convert metadata variable in character
  for(i in 1:length(variables)){
    metadata[, variables[i]] = as.character( metadata[, variables[i]])
  }

#built metadata
x.env = data.frame(SampleID= colnames(x))
  x.env$SampleID= as.character(x.env$SampleID)
  
  for(i in 1:length(variables)){
    x.env$variable= metadata[,variables[i]][match(x.env$SampleID, metadata$SampleID)]
    x.env$variable[is.na(x.env$variable)] = "none"
    x.env$variable[x.env$variable == "NA"] = "none"
    x.env$variable[x.env$variable == ""] = "none"
    colnames(x.env)[colnames(x.env) == "variable"] = paste("variable", i, sep =".")
  }
  
  x.env$mergev = paste( x.env$variable.1,x.env$variable.2, sep ="_" )

  #filter by delivery
  x.env =  x.env[grep(delivery,x.env$mergev),]
  
  #remove NA values in metadata
  sam_to_remove=  x.env$SampleID[ grep("none",x.env$mergev)]
  
  n.samples.x.before = ncol(x)
  
  #remove samples with no information in variables
  if(identical(sam_to_remove, character(0))){
    x.env = x.env
    x = x[, colnames(x) %in% x.env$SampleID]
    
  }else{
    #remove samples with no information for the tested variable
    x.env = x.env[!x.env$SampleID %in% sam_to_remove,]
    x = x[, colnames(x) %in% x.env$SampleID]
    
  }
  
  n.samples.x.after = ncol(x)
  
  if(all(colnames(x)== x.env$SampleID)){
  print("metadata and x are aligned")
  print(paste("no. samples of x: ", n.samples.x.after, " out of ", n.samples.x.before, " selected", sep=""))
  }else{stop("metadata and x are NOT aligned: ERROR")}
  
  
dds <- DESeqDataSetFromMatrix(countData = x,
                              colData = x.env,
                              design= ~ variable.2)


dds <- DESeq(dds)
#resultsNames(dds) # lists the coefficients
res <- results(dds, name= resultsNames(dds)[grep("Intercept", resultsNames(dds),invert =TRUE)])

#convert res as data.frame and add taxonomy

res <- as.data.frame(res)
colnames(res)[1] = paste(colnames(res)[1], resultsNames(dds)[grep("Intercept", resultsNames(dds),invert =TRUE)], sep ="_")

#add sign for the p-value
      
      sig = rep("", nrow(res))
      sig[res$pvalue <= 0.05] <-'.'
      sig[res$pvalue <= 0.01] <-'*'
      sig[res$pvalue <= 0.001] <-'**'
      sig[res$pvalue <= 0.0001] <-'***'    
      
      res$sig = sig

#add sign for the adjusted p-value
res$ad.sig = rep("", nrow(res))

ad.sig = rep("", nrow(res))
      ad.sig[res$padj <= 0.05] <-'.'
      ad.sig[res$padj <= 0.01] <-'*'
      ad.sig[res$padj <= 0.001] <-'**'
      ad.sig[res$padj <= 0.0001] <-'***'

res$ad.sig = ad.sig
return(res)

}

#run an example:

#mystats_2var.p = run_deseq2(x = species_bracken[keep_species,], metadata= metadata,
#                            variables= #c("rand_delivery_method_table","so_eczema_rn_1yr"),delivery = "C-section")


#add column containg taxa information
#mystats_2var.p$taxa = rownames(mystats_2var.p)

#write down table as excel file. It will be needed in the Lefse step.
#convert first element in a list and name is. In this way the name of the object of the list
#will be the name of the sheet of the file. This is useful when you have more than 1 variable
#to analyses

# mystats_2var.p_list = list(mystats_2var.p)
# names(mystats_2var.p_list) = "so_eczema_rn_1yr"
# write.xlsx(mystats_2var.p_list,"Csection_so_eczema_rn_1yr.xlsx")
```

## LEfSe

the LEfSe analysis needs: 1. the read count table; 2. a table where
for each row of 1., the taxonomy is reported; 3. metadata; 4. an excel
file containing the results from DESeq2.The run\_lefse makes use of
bootstraps and thus results might be different for taxa with
moderate/low contributions in microbiome differences.

## LEfSe: steps

1. Obtain read count table. Perform selection of species as for DESeq2
   analyses.

```
species_bracken_select= species_bracken[keep_species,]

#It is important that a column containg the name of the species and their taxID is added. 
#This information will be used in the enxt step when running Lefse analyses
species_bracken_select$taxon = rownames(species_bracken_select)
```

2. Obtain taxonomy table: first, create a function to convert taxonomy
   in the format for lefse analyses (that is each taxonomic level should be
   reported in a different column)

```
convert_tax = function(x){
  mydf= data.frame( Kingdom=NA, Phylum=NA, Class=NA,Order=NA, Family=NA,Genus=NA,Species=NA)
  for(i in 1:length(x)){
    
    Kingdom = strsplit(x[i], split="\\|")[[1]][grep("d__", strsplit(x[i], split="\\|")[[1]])]
    if(identical(Kingdom,character(0))){Kingdom= NA}
    Phylum = strsplit(x[i], split="\\|")[[1]][grep("p__", strsplit(x[i], split="\\|")[[1]])]
    if(identical(Phylum,character(0))){Phylum= NA}
    Class = strsplit(x[i], split="\\|")[[1]][grep("c__", strsplit(x[i], split="\\|")[[1]])]
    if(identical( Class,character(0))){ Class= NA}
    Order= strsplit(x[i], split="\\|")[[1]][grep("o__", strsplit(x[i], split="\\|")[[1]])]
    if(identical(  Order,character(0))){  Order= NA}
    Family = strsplit(x[i], split="\\|")[[1]][grep("f__", strsplit(x[i], split="\\|")[[1]])]
    if(identical(  Family,character(0))){ Family= NA}
    Genus = strsplit(x[i], split="\\|")[[1]][grep("g__", strsplit(x[i], split="\\|")[[1]])]
    if(identical( Genus,character(0))){ Genus= NA}
    Species = strsplit(x[i], split="\\|")[[1]][grep("s__", strsplit(x[i], split="\\|")[[1]])]
    if(identical( Species,character(0))){ Species= NA}
    
    df= data.frame( Kingdom, Phylum, Class,Order, Family,Genus,Species)
    
    mydf= rbind(mydf, df)
  }
  return(mydf[-1,])
}
```

Second, obtain taxonomy from the read count table from
Kraken2/Bracken

```
taxonomy_species = read.delim("bac_vir_fung_counts_species_bracken.csv",
                              sep=",", header = TRUE)[,1:3]
taxonomy_species = taxonomy_species[grep("d__Bacteria",taxonomy_species$Taxonomy),]

taxonomy_species$merged = paste(as.character(taxonomy_species$Species),  as.character(taxonomy_species$TaxID), sep ="_&_") 

taxonomy_species = cbind(taxonomy_species, convert_tax(as.character(taxonomy_species$Taxonomy)))
rownames(taxonomy_species) =NULL 

# add label for plotting 
taxonomy_species$phyl_fa_spec = as.character(unlist(sapply(as.character(taxonomy_species$Taxonomy),
                                                           function(x){
                                                             phylum = strsplit(x, split="\\|")[[1]][grep("p__", strsplit(x, split="\\|")[[1]])]
                                                             phylum = gsub("p__", "", phylum)
                                                             
                                                             family = strsplit(x, split="\\|")[[1]][grep("f__", strsplit(x, split="\\|")[[1]])]
                                                             family = gsub("f__", "", family )
                                                             
                                                             species = strsplit(x, split="\\|")[[1]][grep("s__", strsplit(x, split="\\|")[[1]])]
                                                             species = gsub("s__", "", species )
                                                             
                                                             name <- paste(phylum, family, species, sep= ": ")
                                                             return(name)
                                                             
                                                           })))
```

3. and 4. were already performed above.

Here the function to run LEfSe analyses:

```
run_analyses = function(mag_mat, tax_mat,samples_df,
                        variable,  delivery, wilcoxon_cutoff, norm,kw_cutoff,
                        lda_cutoff,enrich_group, taxa_rank, taxonomy_ori, deseq_file ){
  
  #meaning arguments
  # mag_mat = counts
  # tax_mat= taxonomy
  # samples_df =metadata
  
  colnames(samples_df)[which(colnames(samples_df)==variable)] = "variable"
  
  ### BUILD PHYLOSEQ OBJECT ###
  #eliminate row names of matrices
  rownames(mag_mat) = NULL
  rownames(tax_mat) = NULL
  rownames(samples_df) = NULL
  
  
  mag_mat <- mag_mat %>%
    tibble::column_to_rownames("taxon")%>%
    as.matrix()
  
  tax_mat <- tax_mat %>% 
    tibble::column_to_rownames("merged")%>%
    as.matrix()
  
  samples_df <- samples_df %>% 
    tibble::column_to_rownames("SampleID") 
  
  MAG = otu_table(mag_mat, taxa_are_rows = TRUE)
  TAX = tax_table(tax_mat)
  samples = sample_data(samples_df)
  
  ## phyloseq object ##
  my_phyloseq <- phyloseq(MAG, TAX, samples)
  
  ## choose selected samples according to metadata table ##
  my_phyloseq <- subset_samples(my_phyloseq, select_correct == "yes")
  
  #check if counts are alinged with metadata
  #
  OTU1 = as(otu_table(my_phyloseq), "matrix")
  samples1 = as(sample_data(my_phyloseq), "matrix")
  
  if(all(colnames(OTU1) == rownames(samples1 ))){
    print("counts are aligned with metadata")
  }else{stop("error: counts are NOT aligned with metadata ")}
  
  ## get rid of excess ##
  rm(mag_mat, samples, samples_df, tax_mat, MAG, TAX, OTU1, samples1)
  
  #run lefse using CPM as norm
  #"CPM": pre-sample normalization of the sum of the values to 1e+06
  marker_table <-run_lefse(ps = ps_filter(my_phyloseq, variable != "NA",rand_delivery_method_table==delivery),
                           wilcoxon_cutoff = wilcoxon_cutoff, norm = norm,kw_cutoff = kw_cutoff,lda_cutoff = lda_cutoff,
                           group = "variable", taxa_rank = taxa_rank)
  
  #re-arrange table and add info deseq2
  #convert
  marker_table= as.data.frame(marker_table@marker_table)
  
  if(nrow(marker_table)== 0){
    marker_table = data.frame(taxa = "no marker", values = "no marker")
    return(marker_table)
  }else{
    #replace sign of LDA
    marker_table$ef_lda[marker_table$enrich_group == enrich_group] = -1*marker_table$ef_lda[marker_table$enrich_group == enrich_group]
    #order LDA values
    marker_table = marker_table[order(marker_table$ef_lda, decreasing = TRUE),]
    #built label
    marker_table$label = taxonomy_ori[,grep("phyl_fa", colnames(taxonomy_ori))][match(marker_table$feature,taxonomy_ori[,taxa_rank])] 
    #build order
    marker_table$order = 1:nrow(marker_table)
    #convert in factors group names
    marker_table$enrich_group = as.factor(marker_table$enrich_group)
    #add taxonomy key for deseq2
    marker_table$label_key = taxonomy_ori[,"merged"][match(marker_table$feature,taxonomy_ori[,taxa_rank])] 
    
    
    #add information from deseq2 results
    
    deseq= read_excel(deseq_file, sheet = variable)
    deseq = as.data.frame(deseq )
    deseq = deseq[deseq$ad.sig != "",]
    deseq = deseq[!is.na(deseq$ad.sig),]
    
    #paste sign in labels
    marker_table$ad.sig = deseq$ad.sig[match(marker_table$label_key,deseq$taxa)]
    marker_table$ad.sig[!is.na(marker_table$ad.sig)] ="*"
    marker_table$ad.sig[is.na(marker_table$ad.sig)]= ""
    
    class(marker_table) = "data.frame"
    marker_table = merge(marker_table,deseq, by.x= "label_key", by.y = "taxa", all.x = TRUE,
                         sort = FALSE)
    
    #reorder again according to LDA
    marker_table = marker_table[order(marker_table$ef_lda, decreasing =TRUE),]
    
    
    return(marker_table)
  }
  
}
```

Here an example from the CS-born infants with and without eczema
diagnosed by nurse:

```
metadata$so_eczema_rn_1yr = as.numeric(as.character(metadata$so_eczema_rn_1yr))

csec_nurse_diag_lefse = run_analyses(mag_mat = species_bracken_select,
             tax_mat= taxonomy_species[keep_species,c("merged","Kingdom", "Phylum", "Class","Order", "Family","Genus","Species")],
             samples_df=  metadata[metadata$SampleID %in% mysamples,],
             variable = "so_eczema_rn_1yr",
             delivery = "C-section",
             wilcoxon_cutoff = 0.05,
             norm = "CPM",
             kw_cutoff = 0.05,
             lda_cutoff = 2,
             taxa_rank = "Species",
             enrich_group= 0,
             taxonomy_ori = taxonomy_species,
             deseq_file = "Csection_so_eczema_rn_1yr.xlsx")
```

```
## [1] "counts are aligned with metadata"
```

## Function to plot Lefse results

```
mybar_plot = function(x, variable, delivery){
  
  if(x[1,1] =="no marker" ){
    set.seed(151)
    x<-rnorm(20)
    y<-rnorm(20,1,0.5)
    df<-data.frame(x,y)
    
    myplot= ggplot(df,aes(x,y))+geom_blank()+
      labs(y="LDA Score (log 10)", x=NULL, caption = paste(delivery, variable, sep =", "))
  }else{
    
    #add to sign to label
    x$label_sign =  x$ad.sig.y
    x$label_sign[!is.na(x$label_sign)] = "*"
    x$label_sign[is.na(x$label_sign)] = ""
    
    x$label = paste(x$label, x$label_sign, sep =" ")
    
    
    myplot<- ggplot(x,aes(x=order,y=ef_lda,fill = enrich_group))+
      geom_bar(stat = "identity")+
      labs(y="LDA Score (log 10)", x=NULL, caption = paste(delivery, variable, sep =", "))+
      scale_y_continuous(breaks = seq(-6,6, by=2), limits = c(-6.5, 6.5))+
      theme_minimal() +
      coord_flip()+
      geom_text(aes(y = 0, label = label, hjust = ifelse(ef_lda < 0, -0.03, 1.03)),
                size=3) +theme(axis.text.y = element_blank(),
                               axis.ticks.y = element_blank(),
                               axis.title.y = element_blank(),
                               axis.text.x = element_text(size = 10, face = "bold", colour = "black"),
                               axis.title.x = element_text(size = 13),
                               plot.title = element_text(hjust = 0.5,vjust = -5),
                               legend.position = "top",
                               legend.justification = 0.05,
                               legend.title = element_blank(),
                               panel.grid.major.y = element_blank(),
                               panel.grid.minor.y = element_blank(),
                               panel.grid.major.x = element_line(colour = "grey80", linetype = "dashed"),
                               panel.grid.minor.x = element_blank(),
                               plot.background = element_rect(),
                               plot.caption = element_text(face = "italic", size = 11),
                )
    
    
  }

  return(myplot)
}
```

## Lefse analyses performed on the metabolic pathways

For this analyses, the same tables as for differentially abundant
species are needed. For the taxonomy table, in this case, create a table
containing a first column with the name of the pathways. Copy this
column in a second and label it as “Species”. The function to run the
lefse analyses needs this table. The run\_lefse makes use of bootstraps
and thus results might be different for pathways with moderate/low
contributions in microbiome differences.

```
#read count table
pathways =read.delim("pathway_abund_cpm.csv", sep=",", header = TRUE)
colnames(pathways)[1] = "taxon"

#correct colnames
colnames(pathways) = gsub("\\.", "-", colnames(pathways))
colnames(pathways) = gsub("X", "", colnames(pathways))

# create a table for "taxonomy"
taxonomy = data.frame(taxon = as.character(unlist(sapply(pathways$taxon, function(x) gsub("\\|", "__",x)))))
taxonomy$Species =  pathways$taxon

#remove NA
pathways[is.na(pathways )] = 0

pathways_select = pathways[,colnames(pathways) %in% c("taxon",mysamples)]

#Function to run lefse analyses on metabolic pathways
run_path_analyses = function(mag_mat, tax_mat,samples_df,
                        variable,  delivery, wilcoxon_cutoff, norm,kw_cutoff,
                        lda_cutoff,enrich_group, taxa_rank ){
  
  #meaning arguments
  # mag_mat = counts
  # tax_mat= taxonomy
  # samples_df =metadata
  
  colnames(samples_df)[which(colnames(samples_df)==variable)] = "variable"
  
  ### BUILD PHYLOSEQ OBJECT ###
  #eliminate row names of matrices
  rownames(mag_mat) = NULL
  rownames(tax_mat) = NULL
  rownames(samples_df) = NULL
  
  
  mag_mat <- mag_mat %>%
    tibble::column_to_rownames("taxon")%>%
    as.matrix()
  
  tax_mat <- tax_mat %>% 
    tibble::column_to_rownames("taxon")%>%
    as.matrix()
  
  samples_df <- samples_df %>% 
    tibble::column_to_rownames("SampleID") 
  
  MAG = otu_table(mag_mat, taxa_are_rows = TRUE)
  TAX = tax_table(tax_mat)
  samples = sample_data(samples_df)
  
  ## phyloseq object ##
  my_phyloseq <- phyloseq(MAG,TAX, samples)
  
  ## choose selected samples according to metadata table ##
  my_phyloseq <- subset_samples(my_phyloseq, select_correct == "yes")
  
  #check if counts are alinged with metadata
  #
  OTU1 = as(otu_table(my_phyloseq), "matrix")
  samples1 = as(sample_data(my_phyloseq), "matrix")
  
  if(all(colnames(OTU1) == rownames(samples1 ))){
    print("counts are aligned with metadata")
  }else{stop("error: counts are NOT aligned with metadata ")}
  
  ## get rid of excess ##
  rm(mag_mat, samples, samples_df, tax_mat, MAG, TAX, OTU1, samples1)
  
  #run lefse using CPM as norm
  #"CPM": pre-sample normalization of the sum of the values to 1e+06
  marker_table <-run_lefse(ps = ps_filter(my_phyloseq, variable != "NA",rand_delivery_method_table==delivery),
                           wilcoxon_cutoff = wilcoxon_cutoff, norm =norm,kw_cutoff = kw_cutoff,lda_cutoff = 1,
                           group = "variable", taxa_rank = taxa_rank)
  
  #re-arrange table and add info deseq2
  #convert
  marker_table= as.data.frame(marker_table@marker_table)
  
  if(nrow(marker_table)== 0){
    marker_table = data.frame(taxa = "no marker", values = "no marker")
    return(marker_table)
  }else{
    #replace sign of LDA
    marker_table$ef_lda[marker_table$enrich_group == enrich_group] = -1*marker_table$ef_lda[marker_table$enrich_group == enrich_group]
    #order LDA values
    marker_table = marker_table[order(marker_table$ef_lda, decreasing = TRUE),]
    #built label
    marker_table$label = marker_table$feature
    #build order
    marker_table$order = 1:nrow(marker_table)
    #convert in factors group names
    marker_table$enrich_group = as.factor(marker_table$enrich_group)
    
    class(marker_table) = "data.frame"
    
    #reorder again according to LDA
    marker_table = marker_table[order(marker_table$ef_lda, decreasing =TRUE),]
    
    
    return(marker_table)
  }
  
}

#here an example:

csection_path = run_path_analyses(mag_mat = pathways_select,
                                                     tax_mat= taxonomy,
                                                     samples_df=  metadata[metadata$SampleID %in% mysamples,],
                                                     variable = "po_eczemaUK_1yr",
                                                     delivery = "C-section",
                                                     wilcoxon_cutoff = 0.05,
                                                     norm = "none",
                                                     kw_cutoff = 0.05,
                                                     lda_cutoff = 1.5,
                                                     taxa_rank = "none",
                                                     enrich_group= 0)
```

```
## [1] "counts are aligned with metadata"
```

```
mybar_path_plot = function(x, variable, delivery){
  
  if(x[1,1] =="no marker" ){
    set.seed(151)
    x<-rnorm(20)
    y<-rnorm(20,1,0.5)
    df<-data.frame(x,y)
    
    myplot= ggplot(df,aes(x,y))+geom_blank()+
      labs(y="LDA Score (log 10)", x=NULL, caption = paste(delivery, variable, sep =", "))
  }else{
    
    x$label = x$feature
    
    myplot<- ggplot(x,aes(x=order,y=ef_lda,fill = enrich_group))+
      geom_bar(stat = "identity")+
      labs(y="LDA Score (log 10)", x=NULL, caption = paste(delivery, variable, sep =", "))+
      scale_y_continuous(breaks = seq(-3,3, by=1), limits = c(-3, 3))+
      theme_minimal() +
      coord_flip()+
      geom_text(aes(y = 0, label = label, hjust = ifelse(ef_lda < 0, -0.03, 1.03)),
                size=3) +theme(axis.text.y = element_blank(),
                               axis.ticks.y = element_blank(),
                               axis.title.y = element_blank(),
                               axis.text.x = element_text(size = 10, face = "bold", colour = "black"),
                               axis.title.x = element_text(size = 13),
                               plot.title = element_text(hjust = 0.5,vjust = -5),
                               legend.position = "top",
                               legend.justification = 0.05,
                               legend.title = element_blank(),
                               panel.grid.major.y = element_blank(),
                               panel.grid.minor.y = element_blank(),
                               panel.grid.major.x = element_line(colour = "grey80", linetype = "dashed"),
                               panel.grid.minor.x = element_blank(),
                               plot.background = element_rect(),
                               plot.caption = element_text(face = "italic", size = 11),
                )

  }

  return(myplot)
}
```

## Ecological indeces computation

Function used to rarefy read counts and compute species richness and
Shannon diversity indeces

```
comp_ind = function(x, mysample, mymetadata){
  require(vegan)
  #x= read counts
  #mysample = sample used for rarefaction
  #mymetadata = metadata 
  
  #set seed
  set.seed(201404041)
  
  y= t(x)
  raremax <- min(rowSums(y))
  print(paste("minimum number of reads: ",raremax, sep =""))
  
  #rarefy counts
  rar = rrarefy(y, sample= mysample)
  rowSums(rar)

  #compute shannon and richness
  
  S= apply(rar, 1, function(y) sum(y>0))
  shannon = diversity(rar, index = "shannon")
  mytab = cbind(richness = S, shannon = shannon)
  
  #compute shannon and richness
  mytab = merge(mymetadata, mytab, by.x= "SampleID", by.y = "row.names", all.y = TRUE, sort = FALSE)
 
   #return results and rarefied read count table
  return(list(mytab,rar))
}
```

## Wilcoxon rank sum test and plotting of ecological indeces

the first function is to compute the statistics; the second one is
for plotting.

```
wilcon_two_variable = function(x, variables, index){
  
  #convert metadata variable in character
  for(i in 1:length(variables)){
    x[, variables[i]] = as.character( x[, variables[i]])
  }
  #label with none the missing values
  
  for(i in 1:length(variables)){
    x$variable= x[,variables[i]]
    x$variable[is.na(x$variable)] = "none"
    x$variable[x$variable == "NA"] = "none"
    x$variable[x$variable == ""] = "none"
    colnames(x)[colnames(x) == "variable"] = paste("variable", i, sep =".")
  }
  
  #merge variables
  x$mergev = paste( x$variable.1,x$variable.2, sep ="_" )
  
  sam_to_remove=  x$SampleID[ grep("none",x$mergev)]
  
  if(identical(sam_to_remove, character(0))){
    x = x
    
  }else{
    #remove samples with no information for the tested variable
    x = x[!x$SampleID %in% sam_to_remove,]
  }
  
  #report no of samples after selection
  n.samples = nrow(x)
  
  #copy index column and label it with index
  x$index = x[,index]
  
  #define terms (e.g. CS-born,eczema vs CS-born,no eczema vs Vaginal eczema etc.)
  myterms = unique(x$mergev)
  
  mydf= data.frame(index = NA, variables = NA,terms = NA, n.samples = NA, W = NA, p.value =NA)
  
  
  for(n in 1:(length(myterms)-1)){
    
    if(n ==1){
      
      list.n1 = list(y1 = x[x$mergev %in% c(myterms[n],myterms[n+1]),],
                     y2 = x[x$mergev %in% c(myterms[n],myterms[n+2]),],
                     y3 = x[x$mergev %in% c(myterms[n],myterms[n+3]),])
      
      for(h in 1:length(list.n1)){
        
        tab.n1 = list.n1[[h]]
        
        mytest = wilcox.test(index~mergev, data =tab.n1, paired = FALSE  )
        myterms.n1 = unique(tab.n1$mergev)
        
        mydf.n1= data.frame(index = index, variables = paste(variables, collapse = "_vs_"),terms = paste( myterms.n1, collapse ="_vs_"),
                            n.samples = nrow( tab.n1), W = mytest$statistic, p.value = mytest$p.value)
        
        rm(mytest)
        
        mydf = rbind(mydf,mydf.n1)
      }
      
    }else if(n == 2){
      
      list.n2 = list(y1 = x[x$mergev %in% c(myterms[n],myterms[n+1]),],
                     y2 = x[x$mergev %in% c(myterms[n],myterms[n+2]),])
      
      for(h in 1:length(list.n2)){
        
        tab.n2 = list.n2[[h]]
        
        mytest = wilcox.test(index~mergev, data =tab.n2, paired = FALSE  )
        myterms.n2 = unique(tab.n2$mergev)
        
        mydf.n2= data.frame(index = index, variables = paste(variables, collapse = "_vs_"),terms = paste( myterms.n2, collapse ="_vs_"),
                            n.samples = nrow( tab.n2), W = mytest$statistic, p.value = mytest$p.value)
        
        rm(mytest)
        
        mydf = rbind(mydf,mydf.n2)
        
      }
    }else if(n == 3){
      
      tab.n3 = x[x$mergev %in% c(myterms[n],myterms[n+1]),]
      
      mytest = wilcox.test(index~mergev, data =tab.n3, paired = FALSE  )
      myterms.n3 = unique(tab.n3$mergev)
      
      mydf.n3= data.frame(index = index, variables = paste(variables, collapse = "_vs_"),terms = paste( myterms.n3, collapse ="_vs_"),
                          n.samples = nrow( tab.n3), W = mytest$statistic, p.value = mytest$p.value)
      
      rm(mytest)
      
      mydf = rbind(mydf,mydf.n3)
      
      
      
    }
  }
  
  mydf = mydf[!is.na(mydf$variables),]
  
  sig = c(rep('',length(mydf$p.value)))
  sig[mydf$p.value<= 0.05] <-'.'
  sig[mydf$p.value <= 0.01] <-'*'
  sig[mydf$p.value <= 0.001] <-'**'
  sig[mydf$p.value<= 0.0001] <-'***'
  
  mydf$sig = sig
  
  return(mydf)
  
}

#plotting


myplots_2var = function(x, variables, index){
  require(ggplot2)
  
  #convert metadata variable in character
  for(i in 1:length(variables)){
    x[, variables[i]] = as.character( x[, variables[i]])
  }
  #label with none the missing values
  
  for(i in 1:length(variables)){
    x$variable= x[,variables[i]]
    x$variable[is.na(x$variable)] = "none"
    x$variable[x$variable == "NA"] = "none"
    x$variable[x$variable == ""] = "none"
    colnames(x)[colnames(x) == "variable"] = paste("variable", i, sep =".")
  }
  
  #merge variables
  x$mergev = paste( x$variable.1,x$variable.2, sep ="_" )
  
  sam_to_remove=  x$SampleID[ grep("none",x$mergev)]
  
  if(identical(sam_to_remove, character(0))){
    x = x
    
  }else{
    #remove samples with no information for the tested variable
    x = x[!x$SampleID %in% sam_to_remove,]
  }
  
  #report no of samples after selection
  n.samples = nrow(x)
  
  #plotting
  mytitle = paste(c(variables, "n.samples=", n.samples , "for", index), collapse =" ")
  
  #copy index column and label it with index
  x$index = x[,index]
  
  myplot = ggplot(x, aes(x = mergev, y = index)) + 
    
    #remove outliers added 7.11.2022
    geom_boxplot(outlier.shape = NA)+
    geom_jitter(aes(alpha = 0.5),shape=16)+
    ggtitle(mytitle)+
    labs(x = paste(variables, collapse =", "), 
         y = colnames(x)[which(colnames(x)== index)])
  
  
  return(myplot)
  
}
```

Here, an example from the CS-born infants with and without eczema
diagnosed by nurse

```
FALSE [1] "minimum number of reads: 38520"
```

```
FALSE      index                                      variables
FALSE W  shannon rand_delivery_method_table_vs_so_eczema_rn_1yr
FALSE W1 shannon rand_delivery_method_table_vs_so_eczema_rn_1yr
FALSE W2 shannon rand_delivery_method_table_vs_so_eczema_rn_1yr
FALSE W3 shannon rand_delivery_method_table_vs_so_eczema_rn_1yr
FALSE W4 shannon rand_delivery_method_table_vs_so_eczema_rn_1yr
FALSE W5 shannon rand_delivery_method_table_vs_so_eczema_rn_1yr
FALSE                         terms n.samples    W    p.value sig
FALSE W    Vaginal_0_vs_C-section_0       306 8346 0.02862915   .
FALSE W1     Vaginal_0_vs_Vaginal_1       249 4079 0.54071767    
FALSE W2   Vaginal_0_vs_C-section_1       237 2374 0.56867014    
FALSE W3   C-section_0_vs_Vaginal_1       129 1538 0.47678398    
FALSE W4 C-section_0_vs_C-section_1       117 1038 0.60088832    
FALSE W5   Vaginal_1_vs_C-section_1        60  425 0.92248804
```

Plotting

```
myplots_2var(x= eco_tab[[1]], variables = c("rand_delivery_method_table","so_eczema_rn_1yr"), index = "shannon")
```

## Most abundant taxa

Function used to plot most abundant taxa and compute Wilcoxon rank
sum test on the relative abundance. The first function is the one used
for plotting. Lines and stars report the p-values of the comparisons.
The second function allows to obtain numerical values of the tests
(e.g. p-value, W stats etc).

```
#plotting
myplots_2var = function(x, metadata, variables, level){
  
  #convert metadata variable in character
  for(i in 1:length(variables)){
    metadata[, variables[i]] = as.character( metadata[, variables[i]])
  }
  
  # built metadata table for PCOA:
  
  x.env = data.frame(SampleID= colnames(x))
  x.env$SampleID= as.character(x.env$SampleID)
  
  for(i in 1:length(variables)){
    x.env$variable= metadata[,variables[i]][match(x.env$SampleID, metadata$SampleID)]
    x.env$variable[is.na(x.env$variable)] = "none"
    x.env$variable[x.env$variable == "NA"] = "none"
    x.env$variable[x.env$variable == ""] = "none"
    colnames(x.env)[colnames(x.env) == "variable"] = paste("variable", i, sep =".")
  }
  
  x.env$mergev = paste( x.env$variable.1,x.env$variable.2, sep ="_" )
  
  #remove NA values in metadata
  n.samples.before = ncol(x)
  sam_to_remove=  x.env$SampleID[ grep("none",x.env$mergev)]
  
  #remove samples with no information in variables
  if(identical(sam_to_remove, character(0))){
    x = x
    x.env = x.env
  }else{
    #remove samples with no information for the tested variable
    x.env = x.env[!x.env$SampleID %in% sam_to_remove,]
    x = x[, -which(colnames(x) %in% sam_to_remove )]
  }
  
  n.samples = ncol(x)
  
  
  #plotting
  mytitle = paste(c(variables, "n.samples=", n.samples), collapse =" ")
  
  
  #create a list to collect plots from diffent taxa:
  
  myplot_list = list()
  
  #order based on the relative abundance: most to least abundant.
  taxa = rownames(x)
  taxa = taxa[order(rowMeans(x), decreasing = TRUE)]
  
  
  for(q in 1:length(taxa)){
    
    #copy taxa column to environ x
    if(all(x.env$SampleID == colnames(x) )){
      x.env$taxa = x[which(rownames(x) == taxa[q]),]
      
    }else{stop("invalid: samples labels and their orders are not the same between x and x.env")}
    
    #create my comparisons
    mycomparisons = list(c("C-section_0","C-section_1" ),
                         c("Vaginal_0","Vaginal_1"), 
                         c("Vaginal_0", "C-section_0"),
                         c("Vaginal_1","C-section_1" ))
    
    x.env$mergev = factor(as.character(x.env$mergev), levels =c("C-section_0","C-section_1","Vaginal_0","Vaginal_1"))
    
    p = ggboxplot(x.env, x = "mergev", y = "taxa",
                  title = mytitle,xlab = paste(variables, collapse =", "),
                  ylab = taxa[q],col = "black", outlier.shape=NA)+
      geom_jitter(aes(alpha = 0.3),shape=19, col ="grey34")
    #add p-value wilcoxon
    p2 = p + stat_compare_means(comparisons = mycomparisons, method = "wilcox.test", label = "p.signif") 
    
    myplot_list[[q]] = p2
    
    names(myplot_list)[q] =taxa[q]
    
  }
  
  return(myplot_list)
}

#statistical tests


mystats_2var = function(x, metadata, variables){
  
  require(rlist)
  
  #convert metadata variable in character
  for(i in 1:length(variables)){
    metadata[, variables[i]] = as.character( metadata[, variables[i]])
  }
  
  # built metadata table
  
  x.env = data.frame(SampleID= colnames(x))
  x.env$SampleID= as.character(x.env$SampleID)
  
  for(i in 1:length(variables)){
    x.env$variable= metadata[,variables[i]][match(x.env$SampleID, metadata$SampleID)]
    x.env$variable[is.na(x.env$variable)] = "none"
    x.env$variable[x.env$variable == "NA"] = "none"
    x.env$variable[x.env$variable == ""] = "none"
    colnames(x.env)[colnames(x.env) == "variable"] = paste("variable", i, sep =".")
  }
  
  x.env$mergev = paste( x.env$variable.1,x.env$variable.2, sep ="_" )
  
  #remove NA values in metadata
  sam_to_remove=  x.env$SampleID[ grep("none",x.env$mergev)]
  
  #remove samples with no information in variables
  if(identical(sam_to_remove, character(0))){
    x = x
    x.env = x.env
  }else{
    #remove samples with no information for the tested variable
    x.env = x.env[!x.env$SampleID %in% sam_to_remove,]
    x = x[, -which(colnames(x) %in% sam_to_remove )]
  }
  
  
  #collect taxa
  taxa = rownames(x)
  
  #define conditions and subset table
  #C-section_1 = CS-born with eczema, C-section_0= CS-born without eczema etc.
  
  mycomp = as.data.frame(matrix(c("C-section_1", "C-section_0",
                                  "Vaginal_1", "Vaginal_0",
                                  "C-section_1","Vaginal_1",
                                  "C-section_0","Vaginal_0"), byrow= TRUE, nrow =4 ))
  
  
  #collect all stats
  
  mylist_stats = list()
  
  #create a loop to run stats for every taxum
  for(j in 1:length(taxa)){
    
    
    #check if colnames of x are aligned with the sequenceID list of metadata
    
    if(all(x.env$SampleID == colnames(x) )){
      x.env$taxa = x[which(rownames(x) == taxa[j]),]
      
    }else{stop("invalid: samples labels and their orders are not the same between x and x.env")}
    
    
    mydf = data.frame(taxa = taxa[j])
    
    for(u in 1:nrow(mycomp)){
      stats= data.frame(p.val.1.2=  wilcox.test(x.env$taxa[x.env$mergev==mycomp[u,1]], x.env$taxa[x.env$mergev==mycomp[u,2]], paired = FALSE)$p.value, 
                        W.stats.1.2 = wilcox.test(x.env$taxa[x.env$mergev==mycomp[u,1]], x.env$taxa[x.env$mergev==mycomp[u,2]], paired = FALSE)$statistic, 
                        
                        median.1 = median(x.env$taxa[x.env$mergev==mycomp[u,1]]),
                        median.2 = median(x.env$taxa[x.env$mergev==mycomp[u,2]]),
                        
                        mean.1 = mean(x.env$taxa[x.env$mergev==mycomp[u,1]]),
                        mean.2 = mean(x.env$taxa[x.env$mergev==mycomp[u,2]]),
                        
                        logtwoFC.1.vs.2 = log(mean(x.env$taxa[x.env$mergev==mycomp[u,1]])+0.000000000000001,2)-
                          log(mean(x.env$taxa[x.env$mergev==mycomp[u,2]])+0.000000000000001,2),
                        
                        n.1 = sum(x.env$taxa[x.env$mergev==mycomp[u,1]]>0),
                        n.2 = sum(x.env$taxa[x.env$mergev==mycomp[u,2]]>0))
      
      #add sign for the p-value
      
      sig = ""
      sig[stats$p.val.1.2 <= 0.05] <-'.'
      sig[stats$p.val.1.2 <= 0.01] <-'*'
      sig[stats$p.val.1.2 <= 0.001] <-'**'
      sig[stats$p.val.1.2 <= 0.0001] <-'***'    
      
      stats$sig.1.2 = sig
      colnames(stats) = gsub("1", mycomp[u,1],colnames(stats))
      colnames(stats) = gsub("2", mycomp[u,2],colnames(stats))
      
      mydf = cbind(mydf, stats)
      rm(stats)
      rm(sig)
      
    }
    
    mylist_stats[[j]] = mydf
  }
  
  final_tab_stats = list.rbind(mylist_stats)  
  return(final_tab_stats)
  
}
```

Example of plotting for Firmicutes phylum

```
phylum_counts =read.delim("phylum_bac_counts_bracken..txt",
                          sep="\t", header = TRUE)

#rearrange colnames and substitute NAs with 0 in the read count table
rearr_function= function(x){
  rownames(x) = paste(as.character(x[,1]),  as.character(x$TaxID), sep ="_&_") 
  x = x[, -which(colnames(x) %in% c(colnames(x)[1], "TaxID", "Taxonomy"))]
  colnames(x) = gsub("\\.", "-", colnames(x))
  colnames(x) = gsub("Counts_no_human_", "", colnames(x))
  colnames(x) = gsub("X", "", colnames(x))
  
  #transform NA in 0s
  x[is.na(x)] = 0
  return(x)
}  
  
phylum_counts =rearr_function(phylum_counts)

#compute proportions

phylum_prop = apply(phylum_counts,2,function(x) (x/sum(x))*100)

#select samples
phylum_prop_select = phylum_prop[,colnames(phylum_prop) %in% mysamples]

#loop to generate plots for UK diagnostic tool and nurse diagnosis

myoutcomes= c("po_eczemaUK_1yr", "so_eczema_rn_1yr")

all_plots_p = list()

for(q in 1:length(myoutcomes)){
  all_plots_p[[q]] = myplots_2var(x = phylum_prop_select, metadata=metadata, 
               variables= c("rand_delivery_method_table",myoutcomes[q]), level= "phylum")
}
```

```
## [1] FALSE
## [1] FALSE
## [1] FALSE
## [1] FALSE
## [1] FALSE
## [1] FALSE
## [1] FALSE
## [1] FALSE
## [1] FALSE
## [1] FALSE
## [1] FALSE
## [1] FALSE
## [1] FALSE
## [1] FALSE
## [1] FALSE
## [1] FALSE
## [1] FALSE
## [1] FALSE
## [1] FALSE
## [1] FALSE
## [1] FALSE
## [1] FALSE
## [1] FALSE
## [1] FALSE
## [1] FALSE
## [1] FALSE
## [1] FALSE
## [1] FALSE
## [1] FALSE
## [1] FALSE
## [1] FALSE
## [1] FALSE
## [1] FALSE
## [1] FALSE
## [1] FALSE
## [1] FALSE
## [1] FALSE
## [1] FALSE
## [1] FALSE
## [1] FALSE
```

```
ex = do.call(c, list(all_plots_p[[1]], all_plots_p[[2]]))

#grep plots for Firmicutes
Firmicutes = ex[grep("Firmicutes_&_1239", names(ex))]

Firmicutes
```

```
## $`Firmicutes_&_1239`
```

```
## 
## $`Firmicutes_&_1239`
```

## Additional script to obtain read count table from Bracken output

This R script was used to get the read count tables containing
information for all the samples. The script reads each output from
Bracken and by looping it constructs the read count table. The end of
the code also reports how the sum of all reads classified to
bacterial/fungal/viral etc species were obtained from each samples. N.B.
the script was slightly re-adapted to read files for the genus, family
and phylum level.

```
# #setwd("/data/projects/p529_ABERRANT/projects/misbair/bracken")
# #read files present in the folder and select reports 
# files  <- list.files(full.names = TRUE)
# files_full_report  <- files[grep("noconf.bracken",files)]
# 
# #grep report from taxonomy
# files_report  <- files[grep("__report",files)]
# 
# 
# #create a list of sample names
# samplenames <- as.character(unlist(sapply(files_full_report, function(x) gsub("\\./","",x ))))
# samplenames <- as.character(unlist(sapply(samplenames, function(x) gsub("\\_noconf.bracken","",x ))))
# 
# #create a table with infos about reports and samples
# mytablesamples= as.data.frame(matrix(NA, nrow= length(samplenames),
#                                      ncol =3))
# 
# colnames(mytablesamples)= c("SampleID", "full_report", "report")
# 
# for(i in 1:length(samplenames)){
#   
#   mytablesamples[i,"SampleID"] = samplenames[i]
#   mytablesamples[i,"full_report"] = as.character(files_full_report[grep(samplenames[i], files_full_report)])
#   mytablesamples[i,"report"] = as.character(files_report[grep(samplenames[i], files_report)])
# }
# 
# rm(i)
# 
# #rearrange tables and put results in a list
# 
# full_report_list <- list()
# 
# for(k in 1:nrow( mytablesamples)){
#   
#   #read table full report containing taxID
#   #chech of the file is empty
#   
#   #read table full report containing taxID
#   fulltab= read.delim(mytablesamples$full_report[k], sep ="\t", header = TRUE)
#   
#   #take taxonomy from report table (matched according to the name of the species)
#   tab= read.delim(mytablesamples$report[k], sep ="\t", header = FALSE)
#   tab$V1 = as.character(tab$V1)
#   tab$V3= as.character(unlist(sapply(tab$V1, function(x) strsplit(x , split= "s__")[[1]][2]))) 
#   
#   
#   #bind taxonomy table
#   fulltab$taxonomy <- tab$V1[match(fulltab$name,tab$V3)]
#   
#   #rearrange table columns and rename
#   fulltab = fulltab[,c("name","taxonomy_id",  "taxonomy","new_est_reads")]
#   
#   colnames(fulltab) = c("Species", "TaxID", "Taxonomy", paste("Counts", mytablesamples$SampleID[k], sep="_"))
#   
#   #put results in a list:
#   
#   full_report_list[[k]] <- fulltab
#   names(full_report_list)[k] <- mytablesamples$SampleID[k]
#   rm(fulltab)
#   if(exists("tab")){rm(tab)}
#   
#   
# }
# 
# rm(k)
# 
# 
# myfinalcounts <- full_report_list[[1]]
# 
# for(n in 2:length(full_report_list)){
#   myfinalcounts <- merge(myfinalcounts,full_report_list[[n]], by=c("Species","TaxID","Taxonomy"), all =TRUE, sort =FALSE )
#   
# }
# 
# write.table(myfinalcounts, "bac_vir_fung_counts_bracken.xls", row.names = FALSE, sep="\t")
# 
# #get counts for all reads mapping to the species level.
# summary_reads = as.data.frame(matrix(rep(NA, ncol(myfinalcounts)-3)))
# rownames(summary_reads) = colnames(myfinalcounts)[4:(ncol(myfinalcounts))]
# 
# mylabels = c("d__Bacteria", "d__Viruses", "d__Eukaryota", "d__Archaea","o__Caudovirales")
# 
# for(u in 1:length(mylabels)){
#   
#   mytab = myfinalcounts[grep(mylabels[u], myfinalcounts$Taxonomy),]
#   #eliminate first tree columns containg the character
#   mytab =  mytab[,!colnames( mytab) %in% c("Species", "TaxID", "Taxonomy")]
#   mytab = as.data.frame(colSums( mytab, na.rm = TRUE))
#   colnames( mytab) = gsub("d__", "", mylabels[u])
#   
#   if(all(rownames(summary_reads)== rownames(mytab))){
#     summary_reads = cbind(summary_reads,mytab)
#   }
#   
#   
#   rm( mytab)
#   rm(u)
# }
# 
# summary_reads$V1 = gsub("Counts_no_human_", "", rownames(summary_reads))
# colnames(summary_reads)[1] = "SampleID"
# 
# write.table(summary_reads, "summary_bac_vir_fung_counts_bracken.xls", row.names = FALSE, sep="\t")
```
