# Supplementary material for: The association between the composition of the early-life intestinal microbiome and eczema in the first year of life
Source: Front Microbiomes. 2023 Mar 16;2:1147082. doi: 10.3389/frmbi.2023.1147082 (PMC12993559; doi:10.3389/frmbi.2023.1147082)
Supplement: Supplementary File 2 — Supplementary figures and tables. [file DataSheet_2.pdf]

Supplementary Figure 1. PCoAs based on species abundance analysed with MetaPhlAn 3. Same as figure 1 of the main manuscript, except that 288 species identified with MetaPhlAn 3 are shown. We excluded participants with missing eczema outcomes for panel B and C. Thus, panels (B.) and (C.) were computed by analysing 96.9% (277/286) and 98.3% (281/286) of species, respectively.

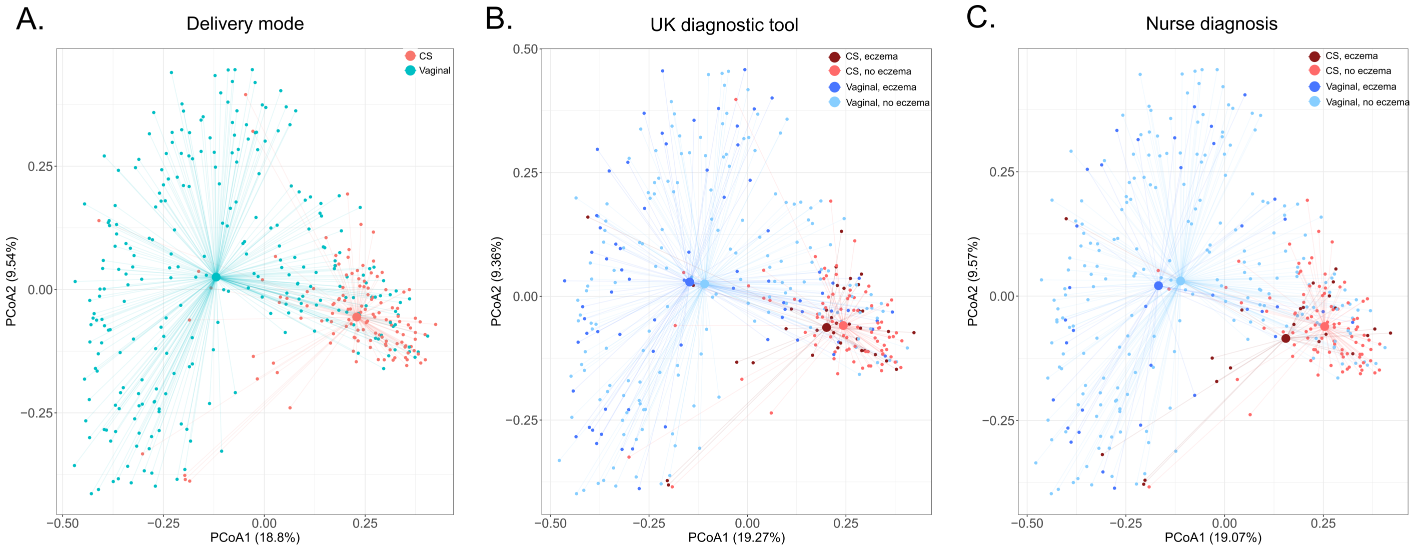

Supplementary Figure 2. Microbiome profiling based on genera abundance according to delivery mode and eczema outcomes. Same as figure 1 of the main manuscript, except that data from 882 genera detected by the Kraken2/Bracken pipeline are shown. We excluded participants with missing eczema outcomes for panel B and C. Thus, panels (B.) and (C.) were computed by analysing 98.6% (870/882) and 99.2% (875/882) of genera, respectively.

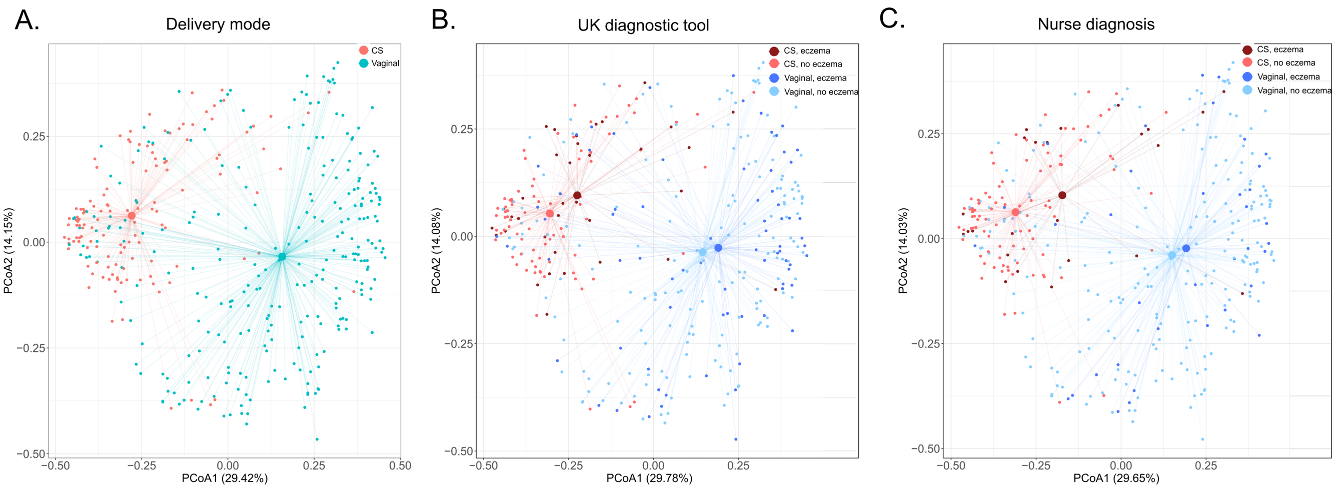

Supplementary Figure 3. PCoA based on genera abundance analysed with MetaPhlAn 3. Same as supplementary figure 2, except that results from 97 genera identified with MetaPhlAn 3 are shown. We excluded participants with missing eczema outcomes for panel B and C. Thus, panels (B.) to (C.) were computed by analysing 99.0% (96/97) of genera, respectively.

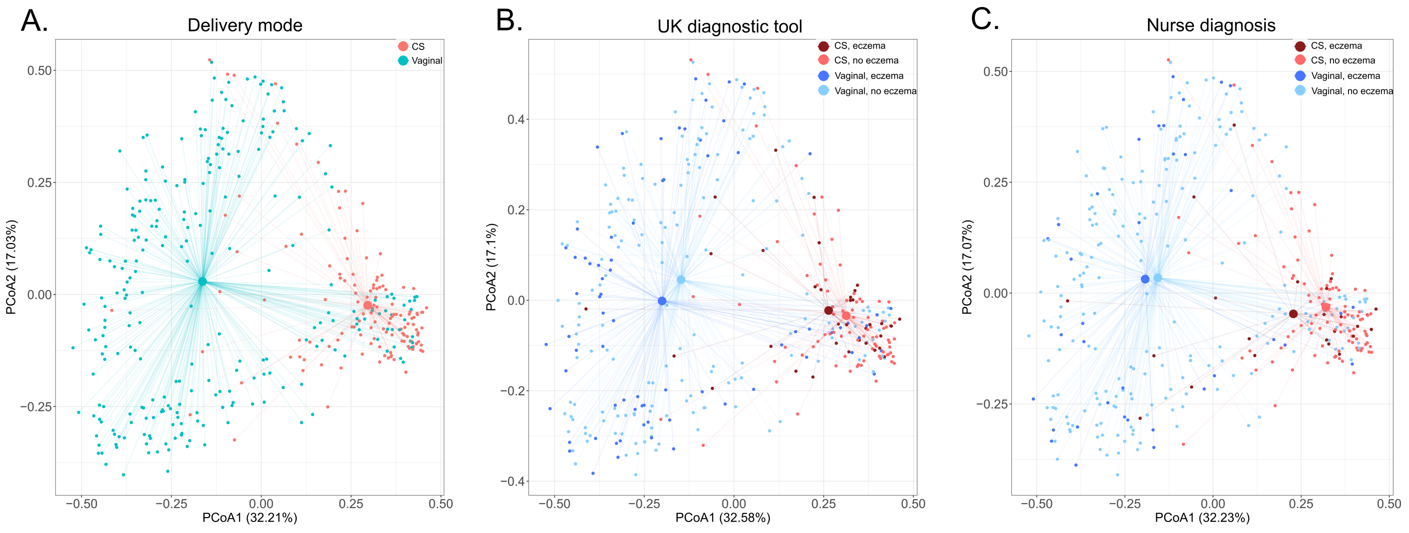

Supplementary Figure 4. Ecological indices. Shannon diversity and richness computed on species identified by the Kraken2/Bracken pipeline. Samples are divided according to delivery mode and eczema outcomes.

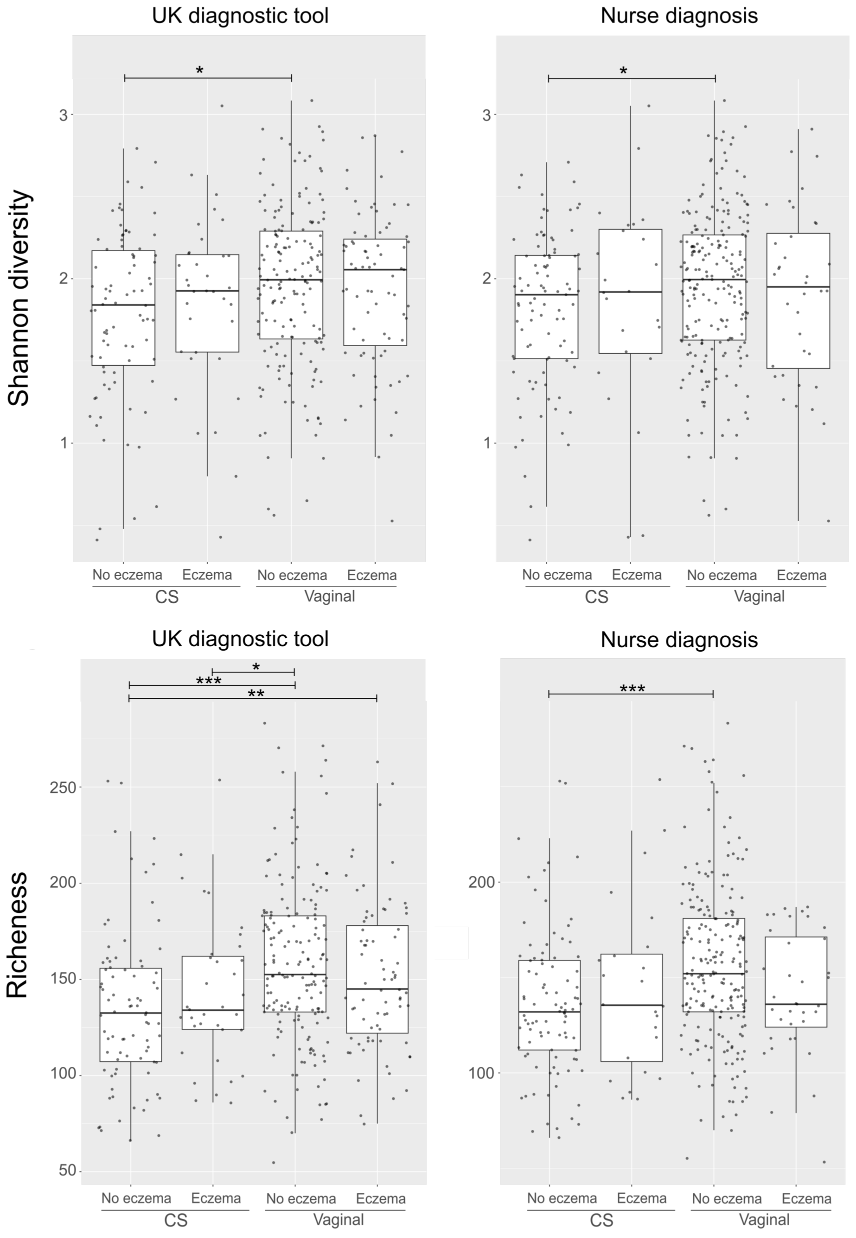

*p<0.05; **p<0.01; ***p<0.0001

Supplementary Figure 5 Distribution of the relative abundance of the four most abundant phyla detected by the Kraken2/Bracken pipeline. Y-axis represents the relative abundance expressed in percentage, x-axis represents the participant group according to delivery mode and eczema outcomes.

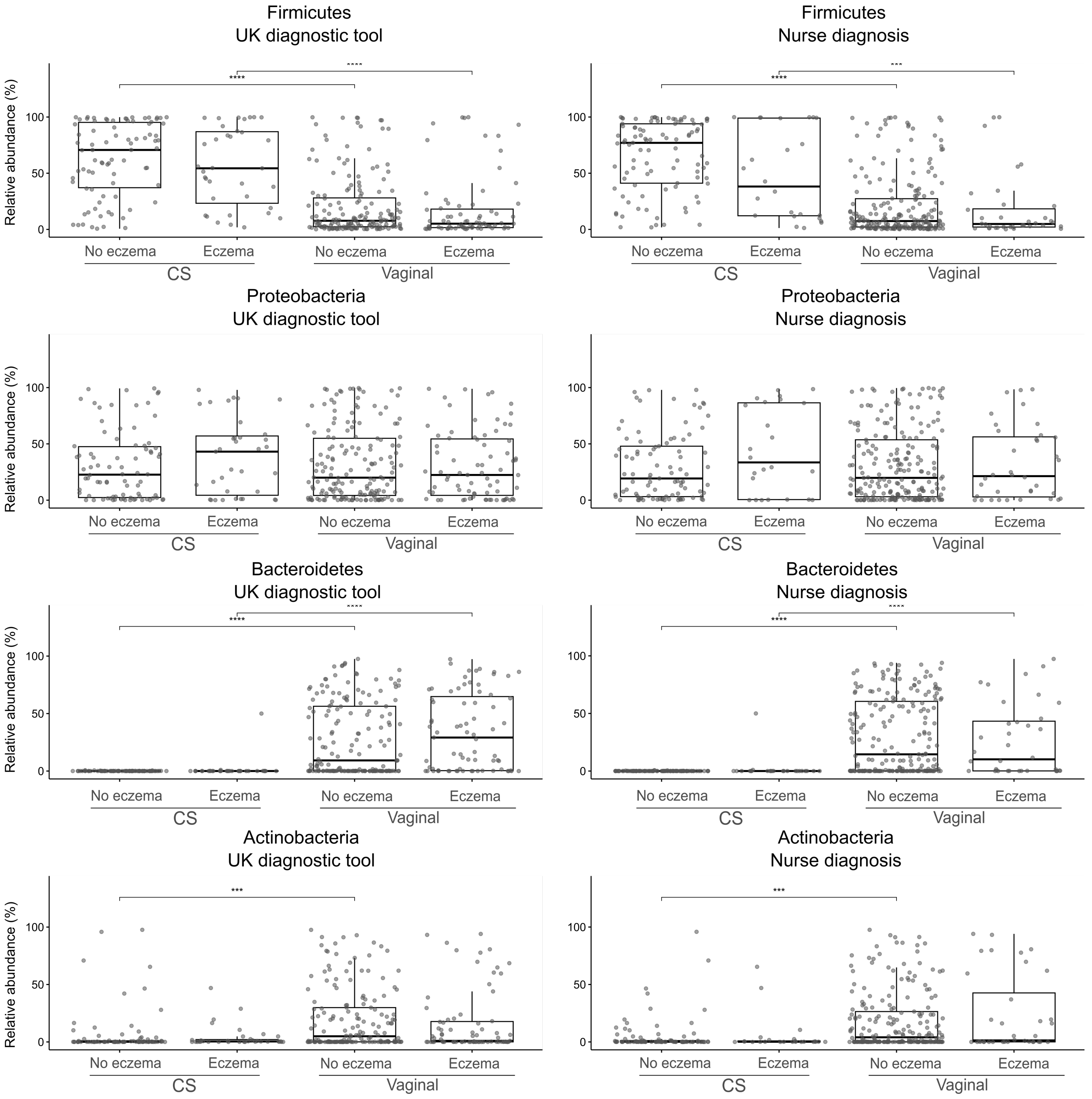

*p<0.05, **p<0.01, ***p<0.001, ****p<0.0001

Supplementary Figure 6. Distribution of the relative abundance of the ten most abundant families detected by the Kraken2/Bracken pipeline. Y-axis represents the relative abundance expressed in percentage, x-axis represents the participant group according to delivery mode and eczema outcomes.

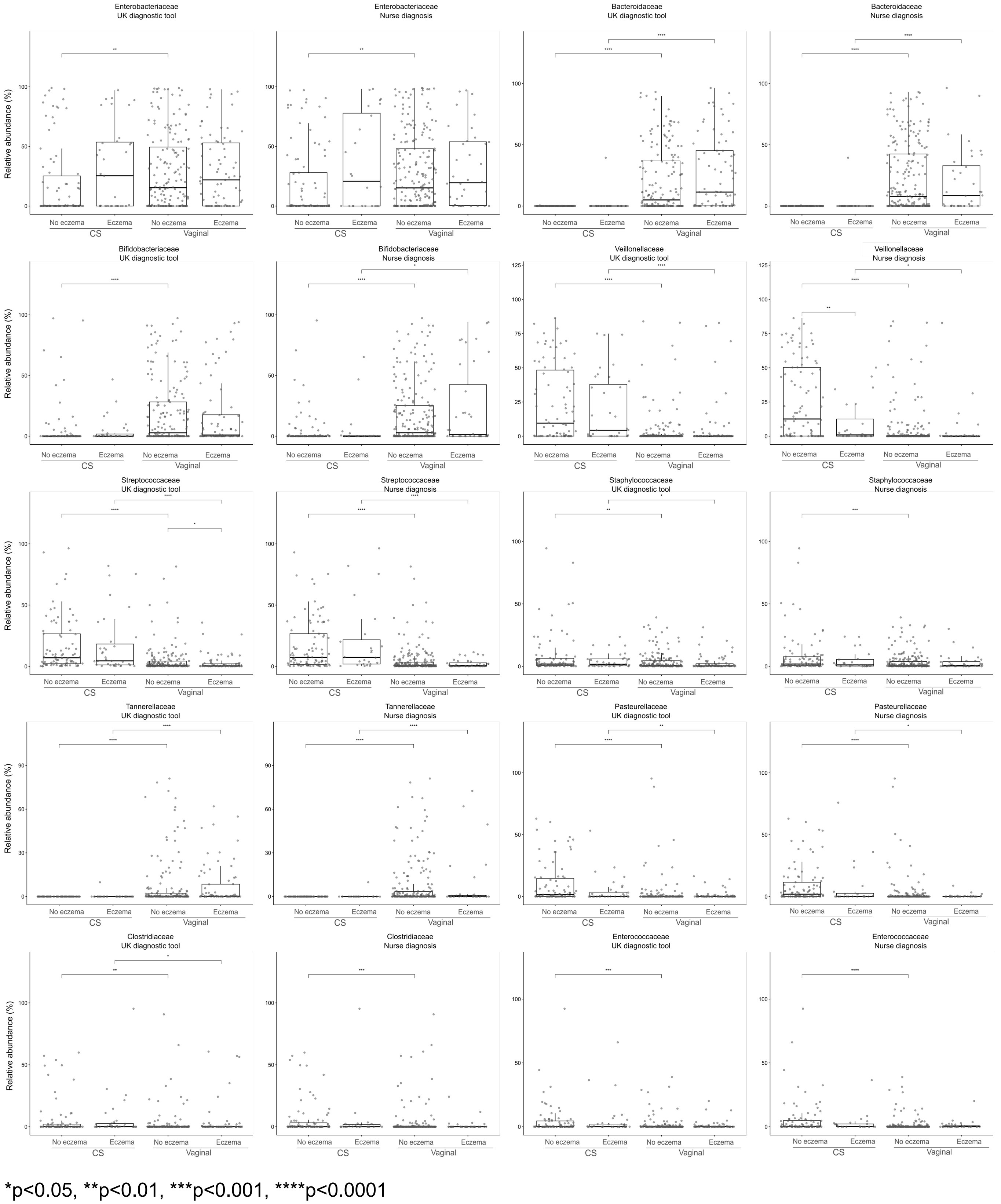

*p<0.05, **p<0.01, ***p<0.001, ****p<0.0001

Supplementary Figure 7. Distribution of the relative abundance of the ten most abundant families detected by the Kraken2/Bracken pipeline. Y-axis represents the relative abundance expressed in percentage, x-axis represents the participant group according to delivery mode and eczema outcomes.

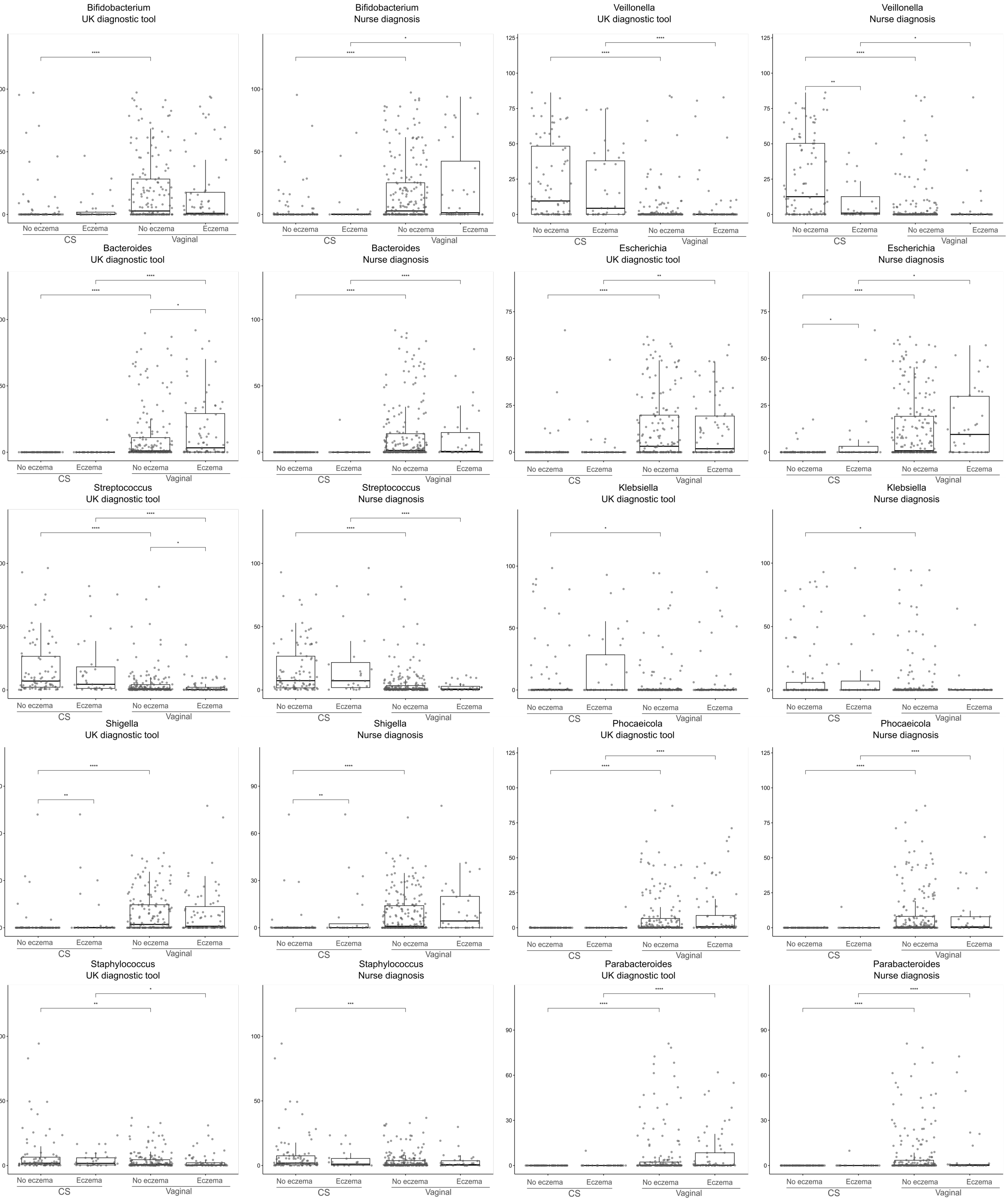

*p<0.05, **p<0.01, ***p<0.001, ****p<0.0001

Supplementary Figure 8. Differentially abundant genera. Same as figure 2 of the main manuscript, except that results run at the genus level are shown. For vaginally born neonates, differentially abundant genera were identified by LEfSe analyses only when eczema was diagnosed with the UK diagnostic tool. *=genera that were also detected differentially abundant with DESeq2 (FDR-adjusted p-value < 0.05). Values are also reported in supplementary file 3.

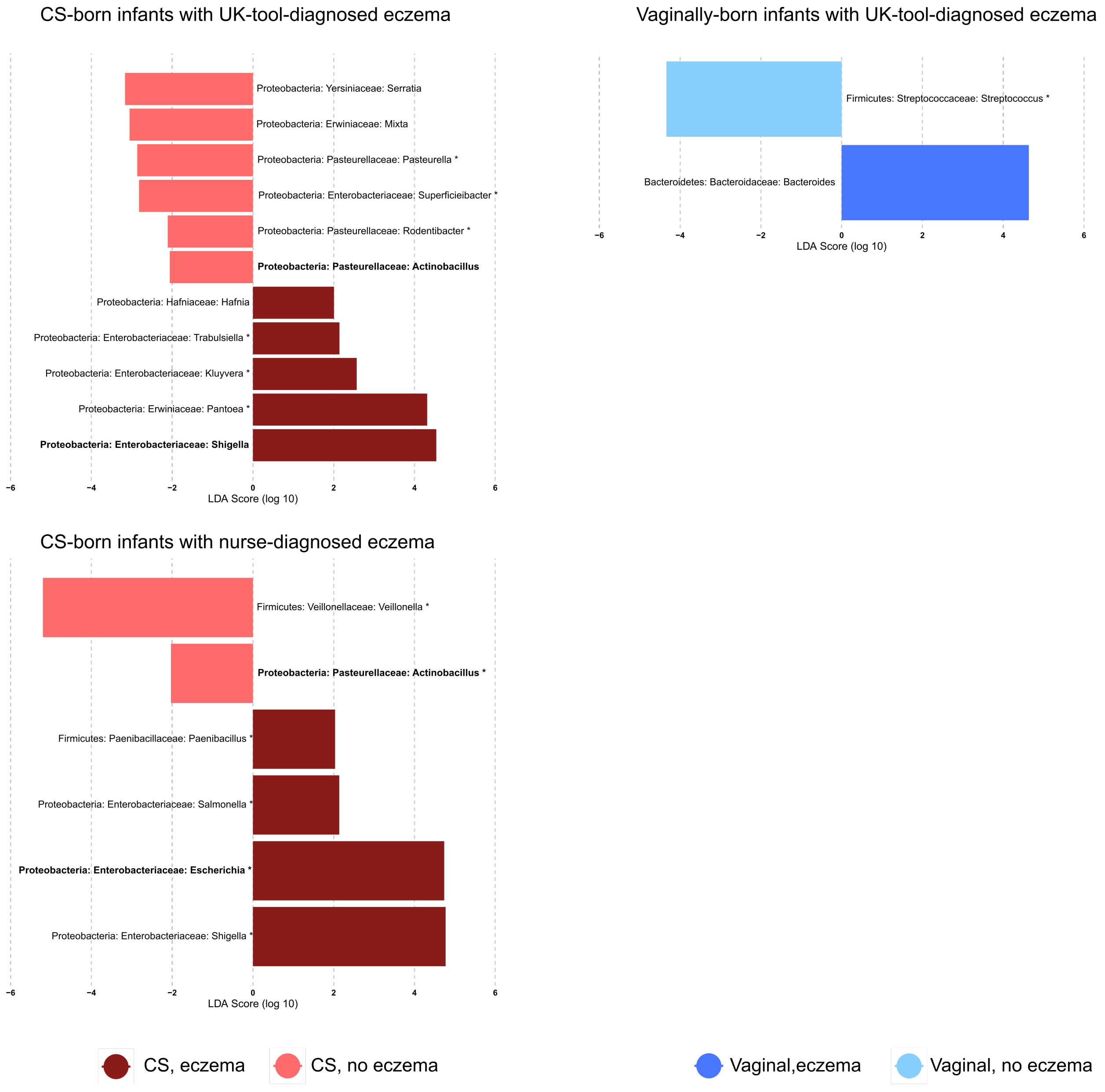

Supplementary Figure 9. Differentially abundant pathways. Same as figure 2 of the main manuscript, except that results run by HUMAnN 3.0 are shown. Values are also reported in supplementary file 3.

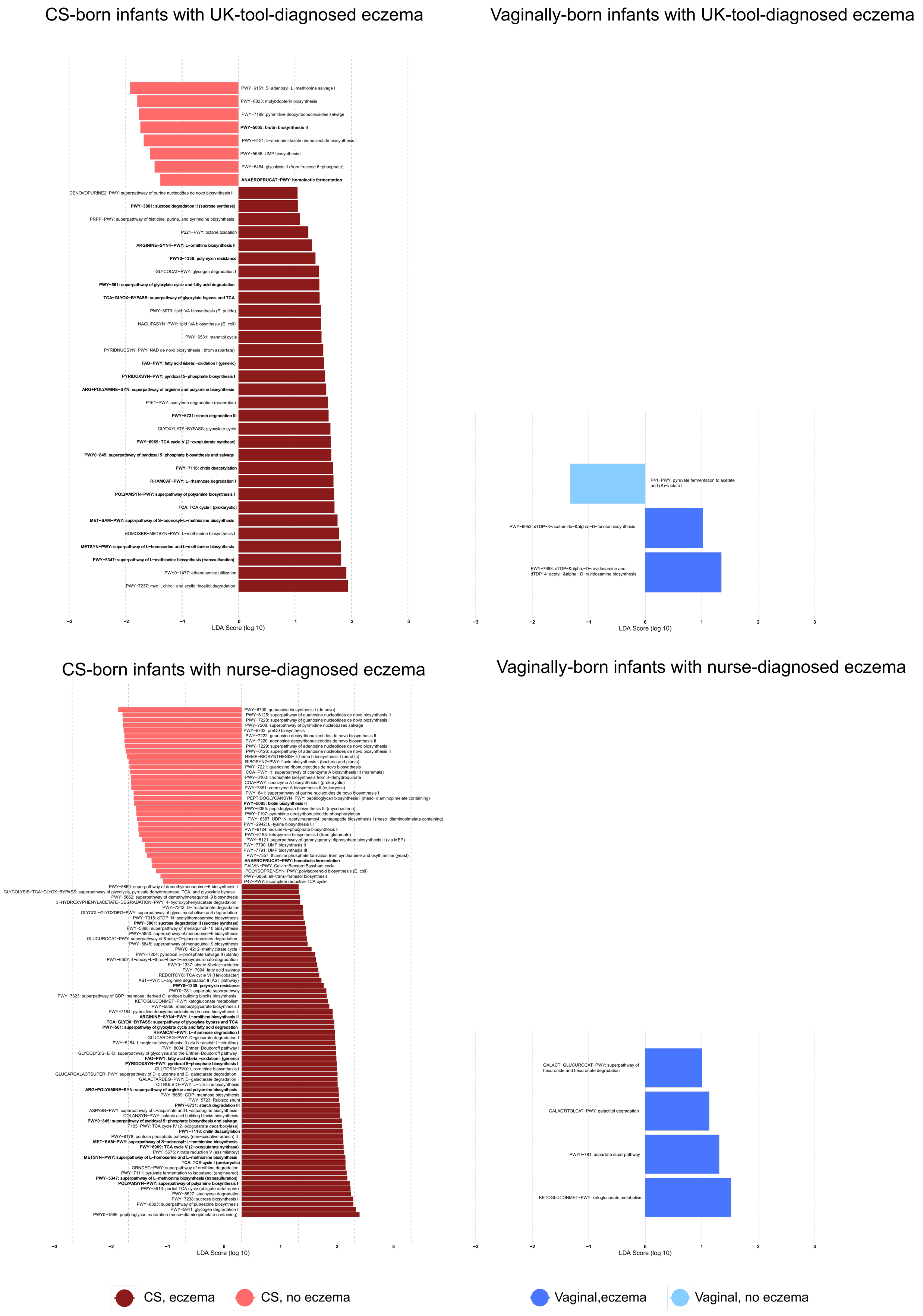

Supplementary Figure 10. Abundance of phages belonging to Caudovirales order. Box plots report the distribution of the relative abundance of phages according to delivery mode and eczema outcomes. Relative abundance is expressed in square-root transformed counts per million.

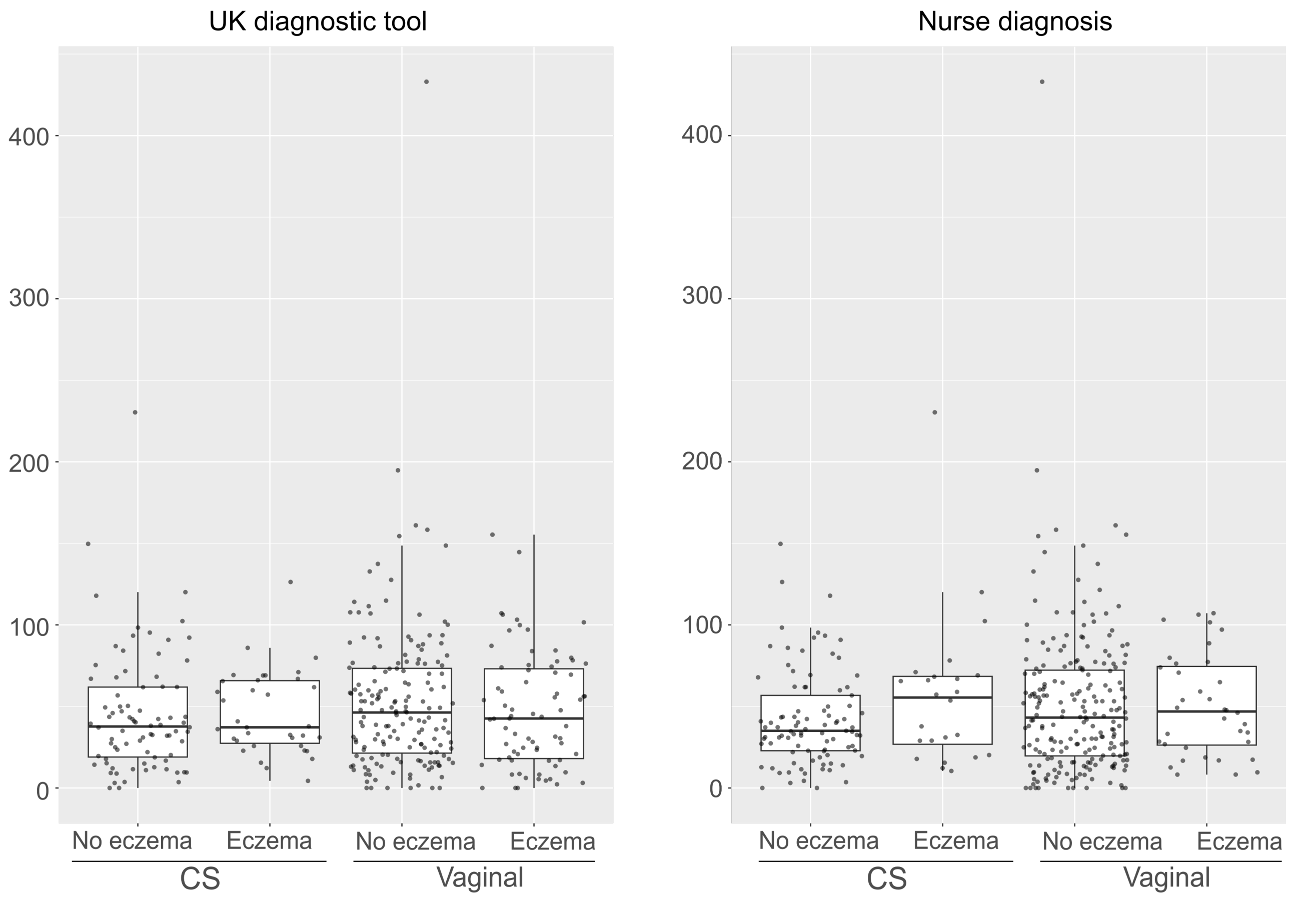

Supplementary Table 1. Association between clinical variables and nurse-diagnosed eczema (chi-square test of independence or simple linear regression).

Factor	p-value
**Infant**	
Delivery mode	0.19
Sex	0.14
Ethnicity	0.15
Gestational age	0.42
Birth weight	0.27
Formula milk before 7 days of life	0.72
Antibiotic exposure in first 12 months of life	0.09
**Maternal**	
Smoking during pregnancy	1.00
Antibiotic use during last trimester of pregnancy	0.24
Antibiotics during labour	0.59
**Environmental**	
Number of siblings	0.86
Childcare attendance in first 12 months of life	0.85
**Animal exposure during pregnancy**	
Pets in household	0.41
**Animal exposure at 12 months of age**	
Pets in household	0.35
**Familial**	
Atopic disease, any parents and/or sibling	0.20
Allergies, any parents and/or sibling	0.24
Atopic disease, both parents	0.17

Supplementary Table 2. Summary of association between intestinal microbiome phylum composition and clinical variables.

Pipeline		Kraken				MetaPhlAn 3			
**Factors**	**No. of samples**	**No. of phyla**	**Pseudo-F**	**P value**	**R2**	**No. of phyla**	**Pseudo-F**	**P value**	**R2**
**Infant**									
Delivery mode	393	19	90.62	**0.0001**	0.1882	5	92.74	**0.0001**	0.1917
Sex	393	19	0.41	0.7051	0.0011	5	0.27	0.7869	0.0007
Ethnicity	393	19	0.98	0.4556	0.0075	5	1.04	0.4048	0.0079
Gestational age	393	19	7.57	**0.0006**	0.0190	5	8.41	**0.0004**	0.0211
Birth weight	393	19	1.36	0.2661	0.0035	5	1.75	0.1681	0.0045
Formula milk before 7 days of life	393	19	8.66	**0.0003**	0.0217	5	8.27	**0.0002**	0.0207
Antibiotic exposure in first 12 months of life	393	19	0.86	0.444	0.0022	5	1.00	0.3813	0.0026
**Maternal**									
Smoking during pregnancy	392	19	2.06	0.12	0.0053	5	2.86	0.0455	0.0073
Antibiotic use during last trimester of pregnancy	393	19	1.89	0.1458	0.0048	5	2.04	0.1265	0.0052
Antibiotics during labour	391	19	6.33	**0.0017**	0.0160	5	8.31	**0.0004**	0.0209
**Environmental**									
Number of siblings	393	19	2.51	**0.005**	0.0252	5	2.48	**0.0072**	0.0250
Childcare attendance in first 12 months of life	390	19	2.29	0.0956	0.0059	5	2.93	**0.0489**	0.0075
**Animal exposure during pregnancy**									
Pets in household	393	19	2.16	0.1066	0.0055	5	2.53	0.0697	0.0064
Cats	393	19	1.10	0.339	0.0028	5	1.22	0.3068	0.0031
Dogs	393	19	1.33	0.2676	0.0034	5	1.34	0.2657	0.0034
Other pets	393	19	1.85	0.1468	0.0047	5	2.42	0.0852	0.0062
Livestock	393	19	0.65	0.5719	0.0017	5	0.68	0.5438	0.0017
**Animal exposure at 12 months of age**									
Pets in household	378	19	1.55	0.2098	0.0041	5	1.62	0.1975	0.0043
Cats	378	19	0.50	0.6491	0.0013	5	0.72	0.5237	0.0019
Dogs	378	19	0.79	0.4757	0.0021	5	0.86	0.4492	0.0023

Other pets	378	19	0.06	0.9184	0.0002	5	0.20	0.8319	0.0005
Livestock	379	19	3.35	0.0258	0.0088	5	3.09	0.0361	0.0081
**Familial**									
Atopic disease, any parents and/or sibling	393	19	0.61	0.5845	0.0015	5	0.77	0.4887	0.0020
Allergies, any parents and/or sibling	393	19	1.03	0.3761	0.0026	5	1.08	0.3587	0.0027
Atopic disease, both parents	393	19	0.41	0.7059	0.0010	5	0.40	0.7001	0.0010
**Eczema outcomes**									
UK diagnostic tool	360	19	1.30	0.2731	0.0036	5	0.89	0.4253	0.0025
Nurse diagnosis	366	19	0.54	0.6249	0.0015	5	0.41	0.6985	0.0011
**Topical steroid use**									
During first 12 months of life	362	19	0.42	0.6984	0.0012	5	0.35	0.7357	0.0009

Supplementary Table 3. Association between clinical variables and the microbiome family composition.

Pipeline		Kraken				MetaPhlAn 3			
**Factor**	**No. of samples**	**No. of families**	**Pseudo-F**	**p-value**	**R2**	**No. of families**	**Pseudo-F**	**p-value**	**R2**
**Infant**									
Delivery mode	393	212	55.39	**0.0001**	0.1241	48	53.91	**0.0001**	0.1212
Sex	393	212	0.51	0.808	0.0013	48	0.39	0.882	0.0010
Ethnicity	393	212	0.88	0.5844	0.0068	48	0.90	0.5403	0.0069
Gestational age	393	212	8.70	**0.0001**	0.0218	48	8.25	**0.0001**	0.0207
Birth weight	393	212	3.59	**0.0052**	0.0091	48	3.18	**0.0083**	0.0081
Formula milk before 7 days of life	393	212	8.09	**0.0001**	0.0202	48	7.71	**0.0001**	0.0193
Antibiotic exposure in first 12 months of life	393	212	1.54	0.1552	0.0039	48	1.70	0.1332	0.0043
**Maternal**									
Smoking during pregnancy	392	212	1.45	0.1887	0.0037	48	1.97	0.078	0.0050
Antibiotic use during last trimester of pregnancy	393	212	1.16	0.3059	0.0029	48	1.21	0.2925	0.0031
Antibiotics during labour	391	212	4.05	**0.0018**	0.0103	48	5.19	**0.0007**	0.0132
**Environmental**									
Number of siblings	393	212	1.85	**0.0096**	0.0187	48	2.07	**0.0043**	0.0209
Childcare attendance in first 12 months of life	390	210	1.71	0.1102	0.0044	48	2.08	0.0681	0.0053
**Animal exposure during pregnancy**									
Pets in household	393	212	1.87	0.0924	0.0048	48	2.34	0.0391	0.0060
Cats	393	212	0.88	0.4852	0.0022	48	1.32	0.2421	0.0034
Dogs	393	212	0.76	0.5812	0.0019	48	0.86	0.4982	0.0022
Other pets	393	212	2.68	**0.022**	0.0068	48	3.01	**0.0125**	0.0076
Livestock	393	212	2.13	0.056	0.0054	48	1.86	0.0851	0.0047
**Animal exposure at 12 months of age**									
Pets in household	378	210	1.42	0.1959	0.0038	48	1.70	0.1255	0.0045
Cats	378	210	0.51	0.8064	0.0013	48	0.66	0.6618	0.0017
Dogs	378	210	0.55	0.7633	0.0014	48	0.79	0.5457	0.0021
Other pets	378	210	0.75	0.5835	0.0020	48	0.92	0.4512	0.0024
Livestock	379	210	1.84	0.0873	0.0049	48	1.59	0.1446	0.0042

**Familial**									
Atopic disease, any parents and/or sibling	393	212	0.59	0.7267	0.0015	48	0.57	0.7378	0.0014
Allergies, any parents and/or sibling	393	212	0.74	0.6003	0.0019	48	0.71	0.6229	0.0018
Atopic disease, both parents	393	212	0.45	0.8462	0.0011	48	0.54	0.76	0.0014
**Eczema outcomes**									
UK diagnostic tool	360	209	1.62	0.1328	0.0045	48	1.34	0.2383	0.0037
Nurse diagnosis	366	210	1.02	0.3824	0.0028	48	0.72	0.6086	0.0020
**Topical steroid use**									
During first 12 months of life	362	210	0.79	0.5666	0.0021	45	0.80	0.5522	0.0022

Supplementary Table 3. Association between clinical variables and the microbiome genera composition.

Pipeline	Kraken					MetaPhlAn 3			
Factor	No. of samples	No. of genera	Pseudo-F	p-value	R2	No. of genera	Pseudo-F	p-value	R2
**Infant**									
Delivery mode	393	882	52.89	**0.0001**	0.1192	97	58.80	**0.0001**	0.1307
Sex	393	882	0.77	0.6171	0.0020	97	0.51	0.8152	0.0013
Ethnicity	393	882	1.17	0.2461	0.0089	97	1.14	0.2986	0.0087
Gestational age	393	882	9.15	**0.0001**	0.0229	97	9.88	**0.0001**	0.0247
Birth weight	393	882	3.93	**0.0012**	0.0010	97	3.75	**0.0032**	0.0095
Formula milk before 7 days of life	393	882	6.70	**0.0001**	0.0169	97	7.09	**0.0001**	0.0178
Antibiotic exposure in first 12 months of life	393	882	1.52	0.1359	0.0039	97	1.77	0.1022	0.0045
**Maternal**									
Smoking during pregnancy	392	882	1.59	0.1125	0.0041	97	1.85	0.0842	0.0047
Antibiotic use during last trimester of pregnancy	393	882	1.12	0.3187	0.0028	97	1.24	0.259	0.0032
Antibiotics during labour	391	882	3.68	**0.0019**	0.0094	97	5.10	**0.0007**	0.0129
**Environmental**									
Number of siblings	393	882	1.58	**0.0289**	0.0160	97	1.88	**0.0063**	0.0190
Childcare attendance in first 12 months of life	390	878	1.37	0.1892	0.0035	97	1.86	0.0845	0.0048
**Animal exposure during pregnancy**									
Pets in household	393	882	1.53	0.1322	0.0039	97	2.15	0.05	0.0054
Cats	393	882	0.87	0.5075	0.0022	97	1.40	0.1944	0.0036
Dogs	393	882	0.78	0.5922	0.0020	97	0.86	0.5088	0.0022
Other pets	393	882	2.48	**0.0198**	0.0063	97	2.83	**0.0126**	0.0072
Livestock	393	882	2.22	**0.0275**	0.0060	97	2.25	**0.0377**	0.0057
**Animal exposure at 12 months of age**									
Pets in household	378	876	1.27	0.2312	0.0034	97	1.69	0.1204	0.0045
Cats	378	876	0.60	0.7852	0.0016	97	0.83	0.5314	0.0022
Dogs	378	876	0.66	0.7264	0.0018	97	0.76	0.5963	0.0020
Other pets	378	876	0.89	0.5035	0.0023	97	0.97	0.4316	0.0026
Livestock	379	876	1.36	0.1899	0.0036	97	1.21	0.2769	0.0032

**Familial**									
Atopic disease, any parents and/or sibling	393	882	0.59	0.7944	0.0015	97	0.58	0.7515	0.0015
Allergies, any parents and/or sibling	393	882	0.73	0.6567	0.0019	97	0.69	0.667	0.0018
Atopic disease, both parents	393	882	0.41	0.9363	0.0011	97	0.57	0.7603	0.0015
**Eczema outcomes**									
UK diagnostic tool	360	870	1.34	0.1969	0.0038	96	1.02	0.3966	0.0029
Nurse diagnosis	366	875	1.05	0.368	0.0029	96	0.85	0.5278	0.0023
**Topical steroid use**									
During first 12 months of life	362	866	0.81	0.5708	0.0022	94	0.81	0.5576	0.0023

Supplementary Table 4. Pairwise PERMANOVA tests according to delivery mode and eczema diagnostic tool at phylum, family, genus and species taxonomic levels.

			Kraken2			MetaPhlAn 3		
**Eczema outcomes**	**Taxonomic level**	**Mode of delivery**	**Pseudo-F**	**p-value**	**R2**	**Pseudo-F**	**p-value**	**R2**
**UK diagnostic tool**	**Phylum**	Vaginally born	0.99	0.3984	0.0041	0.93	0.4248	0.0039
		CS born	1.16	0.2985	0.0010	0.59	0.542	0.0051
	**Family**	Vaginally born	1.01	0.3948	0.0042	1.10	0.3413	0.0045
		CS born	1.66	0.1322	0.0143	1.34	0.232	0.0115
	**Genus**	Vaginally born	0.99	0.4163	0.0041	1.03	0.391	0.0042
		CS born	0.98	0.4494	0.0084	0.88	0.5059	0.0076
	**Species**	Vaginally born	0.56	0.9294	0.0023	0.61	0.8722	0.0025
		CS born	1.15	0.2859	0.0099	0.98	0.4579	0.0085
**Nurse diagnosis**	**Phylum**	Vaginally born	0.31	0.8069	0.0012	0.28	0.8341	0.0011
		CS born	4.23	**0.0285**	0.0355	3.48	**0.0435**	0.0294
	**Family**	Vaginally born	0.49	0.7946	0.0020	0.41	0.8683	0.0016
		CS born	2.95	**0.0144**	0.0250	2.19	**0.0479**	0.0187
	**Genus**	Vaginally born	0.66	0.7134	0.0027	0.61	0.7202	0.0025
		CS born	2.45	**0.0171**	0.0209	2.05	0.0507	0.0175
	**Species**	Vaginally born	0.70	0.7917	0.0028	0.67	0.8069	0.0027
		CS born	1.97	**0.0128**	0.0168	1.45	0.1154	0.0125

CS – Caesarean section
